# Non-decalcified human teeth sample preparation method for MALDI mass spectrometry imaging

**DOI:** 10.1016/j.aca.2026.345386

**Published:** 2026-03-16

**Authors:** Kayle J. Bender, Joanna E. Spurgeon, Chuo Ying Zhai, Manish Arora, Elizabeth K. Neumann

**Affiliations:** a Department of Chemistry, University of California, Davis, One Shields Avenue, Davis, CA, 95616, United States; b Linus Biotechnology, Inc., North Brunswick Township, NJ, 08902, United States; c Icahn School of Medicine at Mount Sinai, New York, NY, 10029, United States

## Abstract

**Background:**

Human teeth preserve a rich temporal record of biological and environmental information throughout an individual’s life. Accessing this record requires analytical techniques capable of providing both spatial and chemical resolution. Matrix-assisted laser desorption/ionization mass spectrometry imaging (MALDI MSI) offers powerful capabilities for visualizing the spatial distribution of biomolecules. However, successful analysis demands preservation of both morphology and chemical integrity. The hard and brittle nature of enamel and dentin presents a major challenge, as preparing sufficiently thin sections typically requires decalcification, which can compromise molecular content.

**Results:**

In this study, we present a sample preparation method using cryofilm, embedding, and specific cryostat blade angles to enable the analysis of non-decalcified human teeth by MALDI MSI. We further demonstrate this approach using various MALDI matrices, highlighting the potential of this technique for spatial analysis of biomolecules in teeth.

**Significance:**

This method provides a foundation for future investigations into the spatial and temporal distribution of small molecules in human teeth.

## GRAPHICAL ABSTRACT

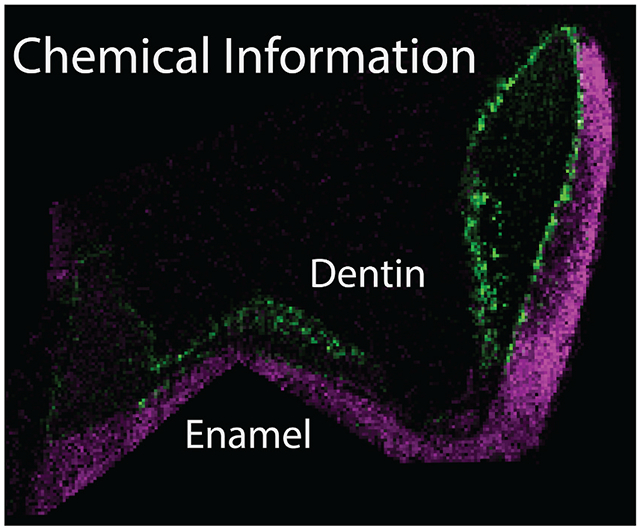

## 1. Introduction

Teeth are mineralized structures composed primarily of enamel, dentin, and the pulp chamber [1]. Enamel, which forms the external surface of the tooth, is recognized as the hardest biological tissue in the human body, primarily due to its high calcium content [2]. Beneath the enamel lies dentin, which has a distinct, layered architecture that develops incrementally over time [3]. The neonatal line is formed at birth within the dentin and enamel and is a histological feature that demarcates prenatal and postnatal growth [4]. These incremental growth layers in dentin are particularly informative, as they entrap molecules circulating in the bloodstream during hydroxyapatite mineralization, providing a temporally resolved chemical archive [5].

Teeth, particularly deciduous teeth shed in early life, have emerged as valuable, non-invasive biospecimens for reconstructing environmental exposure histories. The chemical composition of teeth has been used to assess early-life exposures to metals and other toxicants, with studies linking such exposures to adverse developmental outcomes [6–11]. For instance, nicotine metabolites have been detected in rat dentin following in utero and early postnatal tobacco smoke exposure [12], while prenatal alcohol exposure has been associated with downstream dysregulation in amino acid profiles [11]. Further, altered metal accumulation patterns in teeth have been correlated with neuro-developmental conditions such as autism spectrum disorder [9], and systemic outcomes such as cardiovascular disease linked to poor oral health [13].

Despite their rich temporal record, the utility of teeth in spatially resolved molecular analyses has been limited, because of its hardness. Instead, it is commonly studied after bulk pulverization or significant chemical degradation, resulting in the loss of temporal information encoded within discrete dentin layers [14,15]. Sectioning following decalcification retains spatial information for select proteins but loses small molecule distribution via introduction of EDTA and requires extensive processing times [16]. Embedding and sectioning teeth without decalcification or other treatment preserves the spatial and molecular integrity of the tooth for age-aligned mapping of environmental exposures and endogenous biomolecular content. Thus, spatial analysis of teeth enables retrospective assessment of age-specific exposures and physiological states, particularly when paired with known developmental markers, such as the neonatal line. Aspartic acid, for example, accumulates in a stereospecific manner over time, allowing for age estimation via racemization metrics [17,18]. Proteomic investigations have also identified preserved enamel proteins in archeological samples, revealing the potential for long-term biomolecular stability [19]. These molecular profiles, when preserved and visualized *in situ*, can yield a multidimensional view of developmental history, environmental exposure, and physiological changes.

Matrix-assisted laser desorption/ionization mass spectrometry imaging (MALDI MSI) is uniquely suited for such spatially resolved molecular analyses [20]. In this technique, fresh-frozen biological specimens are cryosectioned to ~10 μm thickness, thaw-mounted onto conductive indium tin oxide (ITO) slides, and coated with a UV-absorbing matrix to facilitate desorption and ionization. A UV laser is rastered across the tissue, generating mass spectra at defined spatial intervals. These data can be reconstructed into ion images at <10 μm, revealing the spatial distribution of metabolites [21–23], lipids [21,24, 25], peptides [26,27], proteins [20,28], and other molecules. While this approach has been extensively applied to soft tissues, the preparation of intact, non-decalcified sections of hard tissues such as human teeth has posed a significant technical challenge due to their rigidity and brittleness [29–31]. Therefore, cryofilm or other tapes are crucial for retaining the spatial integrity of a thin mineralized tissue section. The use of cryofilm for hard tissue sample sectioning has been achieved in previous studies, especially by Kawamoto’s film method [29]. Cryofilm has been extended to mass spectrometry imaging sample preparation for hard tissues such as bone [30,32,33], rat spinal column [31], whole mouse heads [34], whole-body mouse sections [35]. However, teeth pose a unique challenge as the hardest, most calcified biological tissue [2]. The present work aims to overcome the limitations associated with tooth rigidity and brittleness by developing a robust protocol for sectioning non-decalcified, unfixed deciduous and permanent human teeth for MALDI MSI analysis. Here, we show that by using a cryofilm, we can detect numerous features within non-decalcified, unfixed human teeth.

## 2. Materials and methods

### 2.1. Chemicals

All chemicals were purchased from Sigma Aldrich and used without further purification unless otherwise specified.

### 2.2. Tooth collection and sectioning

Six whole teeth (one permanent canine, one permanent molar, one deciduous canine, and three deciduous molars) were donated from deidentified patients and selected based on structural integrity. Patients had no known genetic disorders. Non-decalcified, unfixed human teeth were embedded in 2.6%, 4%, 5.25%, or 7% carboxymethyl cellulose (CMC; EMD Millipore Corp., Burlington, MA) at − 80 °C. The samples were mounted in a Leica CM1860 cryostat microtome (Leica Biosystems, Wetzlar, Germany) at teeth - 20 °C. The cryostat microtome was fitted with the Leica Blade Holder CE-TC at a 0° blade tilt (Leica Biosystems, Wetzlar, Germany). Samples were sectioned up to 10 μm thickness using 30°, 35°, or 40° cutting angle tungsten-carbide blades (SECTION-LAB Co. Ltd., Yokohama Kanagawa, Japan). Teeth were sectioned vertically using Cryofilm type 2C(9) tape (SECTION-LAB Co. Ltd., Yokohama Kanagawa, Japan) to ensure preservation of spatial arrangement of the tooth while sectioning at low temperatures. The ITO-coated glass slide is prepared with copper tape pressed uniformly on the slide using a razor blade, then ZIG 2-way glue is applied on the copper tape. The ZIG 2-way glue is allowed to dry before cryofilm is adhered to the slide and does not directly contact the tooth to avoid glue-related contamination. To adhere cryofilm containing a thin mineralized tissue section to a slide, a PTFE roller chilled to the temperature of the cryomicrotome (− 20 ° C) was used to press the upside-down ITO-coated glass slide against the cryofilm with the adhesive side against the cryomicrotome stage. The cryomicrotome stage was cleaned with methanol and a kimwipe between uses. This strategy compensates for the cryofilm having only one adhesive side, which contains the tissue section. We found that sectioning was successful at varying thickness between 3 and 10 μm. Some teeth were sectioned most successfully as thinner sections (3-5 μm), while others were more successful as thicker sections (5-10 μm) and this tooth-to-tooth variability was empirically determined and not correlated with tooth size or type. We attribute this to the biological variability in human teeth such as the enamel thickness and overall structural integrity. Cryofilm adhered to slide using ZIG 2-way glue (Kuretake Co., Ltd., Nara, Japan) before being heat-fixed at 37 °C.

### 2.3. Staining

Hematoxylin and eosin (H&E) staining (Abcam plc., Cambridge, U. K.) was performed at ~20 °C as previously described [36]. In summary, each tooth section was incubated in hematoxylin, bluing reagent, and then eosin. Slides were prepared with a coverslip using DPX mounting medium (Sigma-Aldrich, Co., St. Louis, MO, USA), then imaged using a ZEISS Axio Scan.Z1 (Brightfield, 2.5 × objective; ZEISS Microscopy, Jena, Germany).

### 2.4. Matrix application

α-Cyano-4-hydroxycinnamic acid (CHCA; 5 mg/mL in 70% methanol; Sigma-Aldrich, Co., St. Louis, MO, USA), 1,5-diaminonaphthalene (DAN; 20 mg/mL in tetrahydrofuran; Tokyo Chemical Industry Co., Ltd., Portland, OR, USA), 2,5-dihydroxyacetophenone (DHA; 10 mg/mL in 70% acetonitrile; Thermo Fisher Scientific, Heysham, England), or 2,5-dihydroxybenzoic acid (DHB; 40 mg/mL in 70% methanol; Sigma- Aldrich, Co., St. Louis, MO, USA) matrices were applied using an HTX M3+ sprayer (HTX Technologies, LLC, Chapel Hill, NC). Spray parameters for each matrix can be found in SI Table S1.

## 3. MALDI Q-TOF MSI

MALDI MSI experiments were performed using a timsTOF fleX dual source MALDI mass spectrometer (Bruker Scientific, Billerica, MA). Experiments were performed in either positive or negative ion mode with a 30 μm × 30 μm raster width. Raster width is used here to describe the scanned area per laser ablation. Teeth were analyzed by MALDI MSI for lipids, peptides, and small molecule metabolites in positive mode, and lipids in negative mode. Lipid and peptide features were analyzed using a MALDI MSI method optimized for lipids. Small molecule metabolite features were analyzed using a MALDI MSI method optimized for metabolites. Each MALDI MSI method was used across CHCA, DAN, DHA, and DHB matrices. MALDI MSI method details are available in Supporting Information. On-tissue MS/MS was performed on tooth serial sections by collision induced dissociation (20-40 eV) with nitrogen gas using a timsTOF fleX mass spectrometer.

### 3.1. Data analysis

Data were visualized and feature finding was performed using SCiLS Lab Version 2025b Pro software (Bruker Scientific). Box plots representing peak intensity of pixels in each region were produced by SCiLS Lab software. Mass spectral features with mass defect between 0.4 and 0.7, between *m/z* 700-900 are putatively annotated as lipids using LIPIDMAPS database and mass accuracy (<5 ppm) [37]. Subsequent on-tissue MS/MS was performed on tooth serial sections for lipid headgroup verification (spectra available in Supporting Information). Pathos web facility was used to facilitate potential metabolite matching to Kyoto Encyclopedia of Genes and Genomes (KEGG), searching for metabolites only in homo sapiens and considering + H, +Na, or + K adducts with 10 ppm mass tolerance [38] with cross referencing using the Human Metabolome Database (HMDB) [39]. MS/MS of potential metabolite features were matched to MassBank using precursor accurate mass, fragmentation accurate mass, and fragmentation relative intensity (table available in Supporting Information) [40]. METLIN database was used to search for potential peptide features (<5 ppm), considering +H, +Na, or +K adducts [41]. Mass spectral features included in database searches had an intensity of at least 1% of the base peak intensity.

## 4. Results and discussion

**Fig. 1.**
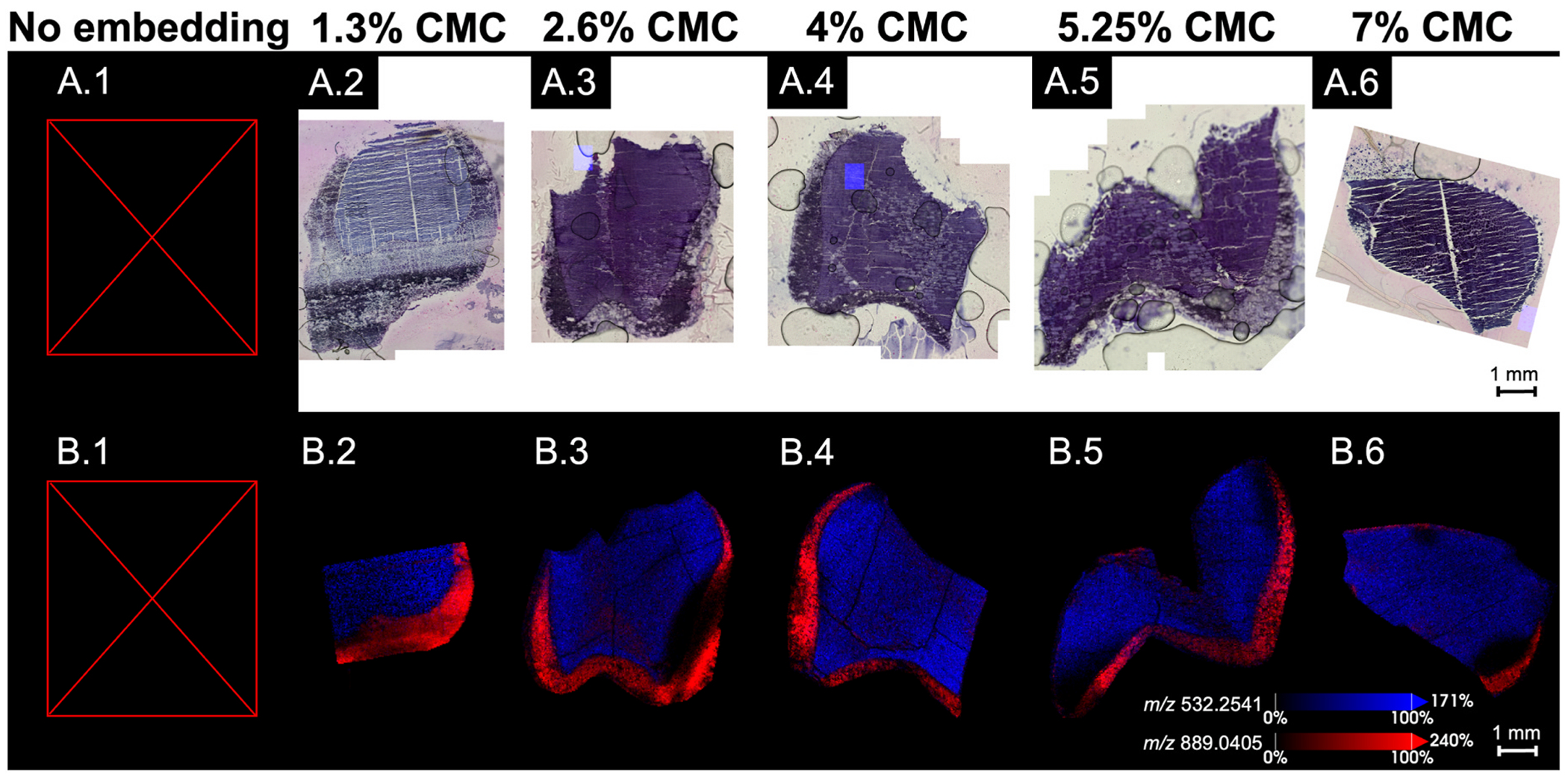
Embedding material aided in sectioning of non-decalcified human teeth. Teeth were unable to be sectioned without embedding material due to tooth shattering or blade breakage (A.1-B.1). Teeth embedded in 1.3%, 2.6%, 4%, 5.25%, or 7% carboxymethylcellulose (CMC) were sectioned and hematoxylin and eosin (H&E) stained (A.2-6, respectively). Subsequent tooth sections were prepared for MALDI MSI (B.2-6). Layered ion images (B.2-6) represent the relative abundance of *m/z* 889.0405 (enamel, red) and an ion putatively annotated as peptide LYAK (*m/z* 532.2541, dentin, blue) according to METLIN database. Crack measurements are available in SI Figure S3. (For interpretation of the references to colour in this figure legend, the reader is referred to the Web version of this article.)

Sectioning of mineralized tissues such as human teeth presents significant mechanical challenges due to their inherent brittleness and heterogeneous composition. Here, we evaluated the role of carboxymethyl cellulose (CMC) concentration in embedding media to determine the minimum concentration required for successful cryosectioning of non-decalcified human teeth. A key observation was that attempts to section teeth without embedding them in CMC consistently resulted in shattering of either the tooth or blade (Fig. 1 A–B.1). In these cases, the cryostat blade frequently deflected or bent around the hard tissue, indicating the absence of sufficient external support during sectioning.

Embedding teeth in CMC provided structural reinforcement, effectively reducing damage to the tooth and blade by distributing applied forces more uniformly. Notably, we found that while increasing the concentration of CMC does improve the rigidity of the embedding matrix, it also substantially increases preparation time and effort. Concentrations higher than 2.6% CMC were more difficult to work with and correlated with an increased incidence of tooth breakage during sectioning, likely because of the rigidity of the embedding medium. The lowest concentration, 1.3% CMC (Fig. 1 A–B.2), also resulted in increased tooth cracking compared to 2.6% CMC (Fig. 1 A–B.3). To evaluate extent to cracking in tooth sections, several of the widest cracks in each section were measured (measurement annotations provided in SI Figure S3). While crack width is a limited analysis of cracking severity, it is a quantifiable indicator of spatial distortion in tooth sections. Teeth embedded in 2.6% CMC had the smallest cracks ranging from 11 to 21 μm. Teeth embedded in 4% CMC (Fig. 1 A–B.4) resulted in slightly more cracking than teeth embedded in 2.6% CMC, with the widest cracks ranging from 23 to 29 μm (SI Figure S3). Furthermore, teeth embedded in 1.3% CMC (Fig. 1 A–B.2) and 5.25% CMC (Fig. 1 A–B.5) had some visible cracking, and teeth embedded in 7% CMC resulted in the most cracking (Fig. 1 A–B.6). The widest cracks in tooth sections embedded 5.25% CMC ranged from 28 to 64 μm, while teeth embedded in 7% CMC resulted in cracks 40 – 129 μm wide. The cracks in tooth sections embedded in 1.3% CMC ranged from 30 to 56 μm, making it most similar to crack width observed with 5.25% CMC. However, the cracks were most numerous in 1.3% and 7% CMC (Fig. 1 A–B.2, Fig. 1 A–B.6, respectively). Based on these findings, 2.6% CMC was identified as the lowest effective concentration that balances ease of preparation with successful sectioning outcomes (Fig. 1 A–B.3). Although 2.6% CMC requires less laborious preparation than 4% and 5.25% CMC, either concentration may also be used for successful sectioning. Regardless, embedding media is required for sectioning.

**Fig. 2.**
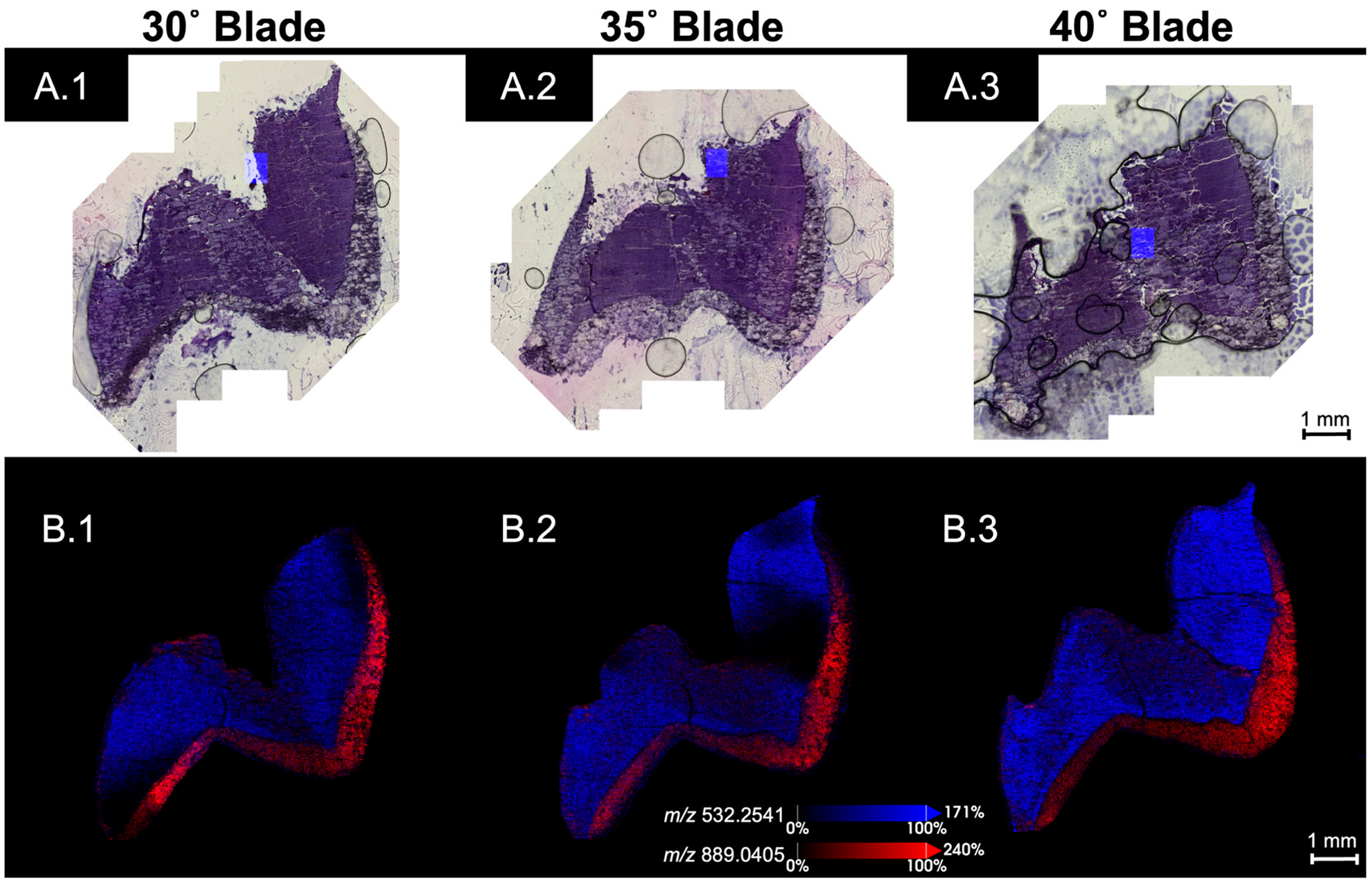
Tungsten-carbide sectioning blades with blade angles of 30° and 35° exhibit reduced section fracturing compared to blades with 40°. Representative hematoxylin and eosin (H&E) stained sections of non-decalcified human teeth were prepared using 30-, 35-, and 40-degree tungsten-carbide blades (A.1-3, respectively). MALDI MSI was performed on subsequent sections of each tooth. Layered ion images (B.1-3) represent the relative abundance of *m/z* 889.0405 (enamel, red) and an ion putatively annotated as peptide LYAK (*m/z* 532.2541, dentin, blue) according to METLIN database. Crack measurements are available in SI Figure S4. (For interpretation of the references to colour in this figure legend, the reader is referred to the Web version of this article.)

Blade selection emerged as a critical factor influencing the success of cryosectioning non-decalcified human teeth. Thinner, disposable tungsten-carbide blades are typically used for soft tissue sectioning but proved unsuitable for this application. These blades either bent around the tooth during attempts to collect thin sections or fractured entirely when thicker sections (>50 μm) were attempted. As a result, we were unable to obtain useable dental sections with the thinner tungstencarbide blades, highlighting the necessity of using more robust tungsten-carbide blades for sectioning teeth. Among the tested blade geometries, SL series tungsten-carbide blades with angles of 30° , 35°, and 40° were all capable of sectioning embedded teeth (Fig. 2). However, the 40° angle consistently produced extensive cracking up to 102 μm wide, which is visibly apparent by microscopy and MALDI MSI (Fig. 2. A.3 and B.3, respectively). In contrast, both 30° and 35° angles yielded cleaner, more consistent sections, with reduced structural disruption (Fig. 2 A–B.1 and A–B.2, respectively). Teeth sectioned using the 30° blade angle resulted in cracks up to 17 μm wide, while the 35° blade angle yielded similar cracks up to 18 μm wide. To maintain section quality and prevent cross-contamination, blades were cleaned with methanol after each use. Blade quality after sustained usage further influenced performance; to address this, a single portion of the blade was designated for initial trimming, while a fresh, unused segment was reserved for collecting sections of interest. This approach ensured both precision and reproducibility in sectioning mineralized tissue.

To evaluate the suitability of MALDI MSI for lipid and other small molecule detection in teeth, we performed preliminary assessments using CHCA, DHB, DHA, and DAN MALDI matrices to determine what molecular features could be detected within the untreated teeth. Teeth were serially sectioned, coated in the four matrices above, and analyzed by MALDI MSI. One experiment targeted lipids in positive mode, the second experiment targeted lipids in negative mode, and the third targeted small molecules in positive mode. A limited number of peaks consistent with lipid species were observed in positive mode lipid analysis, but none were found in negative mode lipid analysis. Interestingly, more features in the positive mode lipid analysis experiment matched the *m/z* value of small peptides, three to five amino acids in length (SI Table S2). DAN matrix resulted in the highest number of potential peptide features, with 62 features matching peptides in the METLIN database by *m/z* value (<5 ppm). Potential peptide features within 5 ppm of features found in the matrix negative control were not considered. We also acknowledge that DAN can induce some fragmentation in source and would explain the number of detected features. Conversely, DHA, DHB, and CHCA had very few features with potential peptides matches (9, 5, and 4, respectively). Unfortunately, peak intensity was too low for sufficient on-tissue tandem MS of these mass spectral features in all four matrices. The low intensity of these features may be indicative of low peptide abundance or inefficient ionization of peptides in the non-decalcified hydroxyapatite crystalline structure. Future MALDI MSI experiments for peptide detection in non-decalcified teeth would benefit from an established workflow for optimizing peptide detection, whereas the potential peptide features herein were observed in an experiment optimized for lipid detection.

**Fig. 3.**
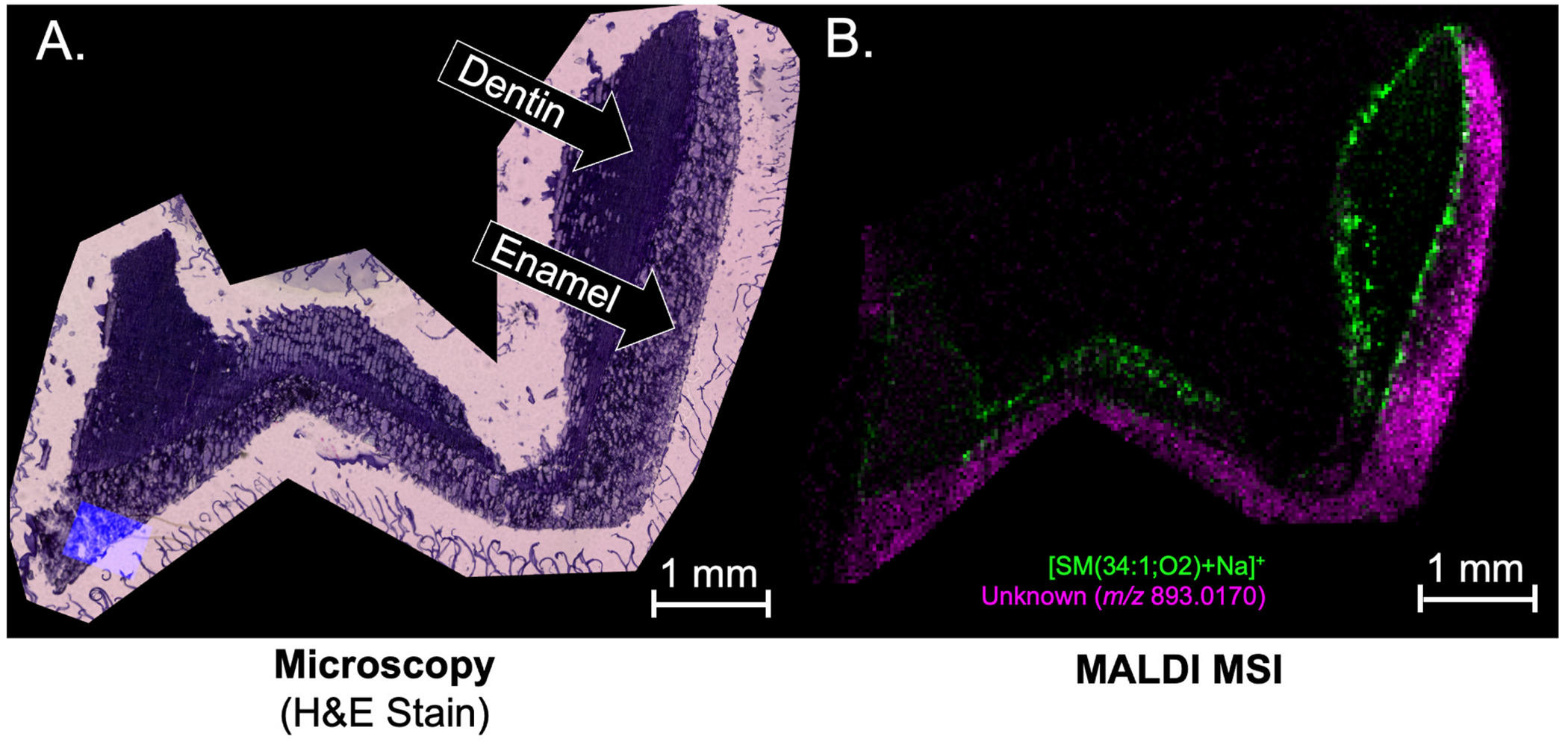
Mass spectral features consistent with lipids were found at the dentin-enamel junction and dentin-pulp interface using CHCA matrix. Anatomy of the tooth is represented by dentin and enamel regions observed by microscopy image of hematoxylin and eosin (H&E) stained tooth section (A). [SM(34:1; O2) + Na]^+^ localized primarily to the dentin-enamel junction and dentin-pulp interface (B).

Among the matrices tested for lipid detection, CHCA and DHB enabled the most promising results because lipid features were only found when using CHCA and DHB. CHCA and DHB also had higher signal to noise ratios (S/N > 2000) at the base peak compared to DAN (S/N 695) or DHA (S/N 236). CHCA enabled detection of [SM(34:1; O2) + H]^+^ and [SM(34:1; O2) + Na]^+^ (SI Figure S5) at the dentin-enamel junction and the interface between dentin and pulp (Fig. 3). The dentin-enamel junction is approximately less than 20 μm, less mineralized than enamel or dentin and contains more organic matter [42–44]. This protective region is recognized as a collagen-rich boundary that joins the dissimilar dentin and enamel, and prevents cracks in enamel from continuing through the dentin [44]. Lipids found in the dentin-enamel junction may be involved in the protective role of the region, as lipids are components of the extracellular matrix in calcified tissues [45]. At the interface of dentin and pulp, there is predentin, which is an unmineralized organic matrix secreted by odontoblasts [46]. Lipids localized here may correspond to sites of active cellular activity, including odontoblast function [47]. Additionally, there were two lipid features, *m/z* 827.4456 (unknown) and *m/z* 843.5299 (unknown), found primarily in enamel when using DHB (SI Figure S6). Unfortunately, *m/z* 827.4456 and *m/z* 843.5299 are both low intensity peaks (<1% of base peak intensity), in which case on-tissue tandem MS would be unreliable. Neither *m/z* 827.4456 nor *m/z* 843.5299 matched the accurate mass of known lipids in LIPID MAPS database. Ultimately, the recommended matrix for positive mode lipid analysis in non-decalcified teeth is CHCA due to the above observations. The limited detection of lipid signals in these regions may reflect both the limited abundance of preserved lipids in mineralized tissues and the difficulty of ionizing lipids in the crystalline tooth microstructures. This line of investigation may ultimately allow for retrospective assessment of lipid presence from systemic circulation during development.

**Fig. 4.**
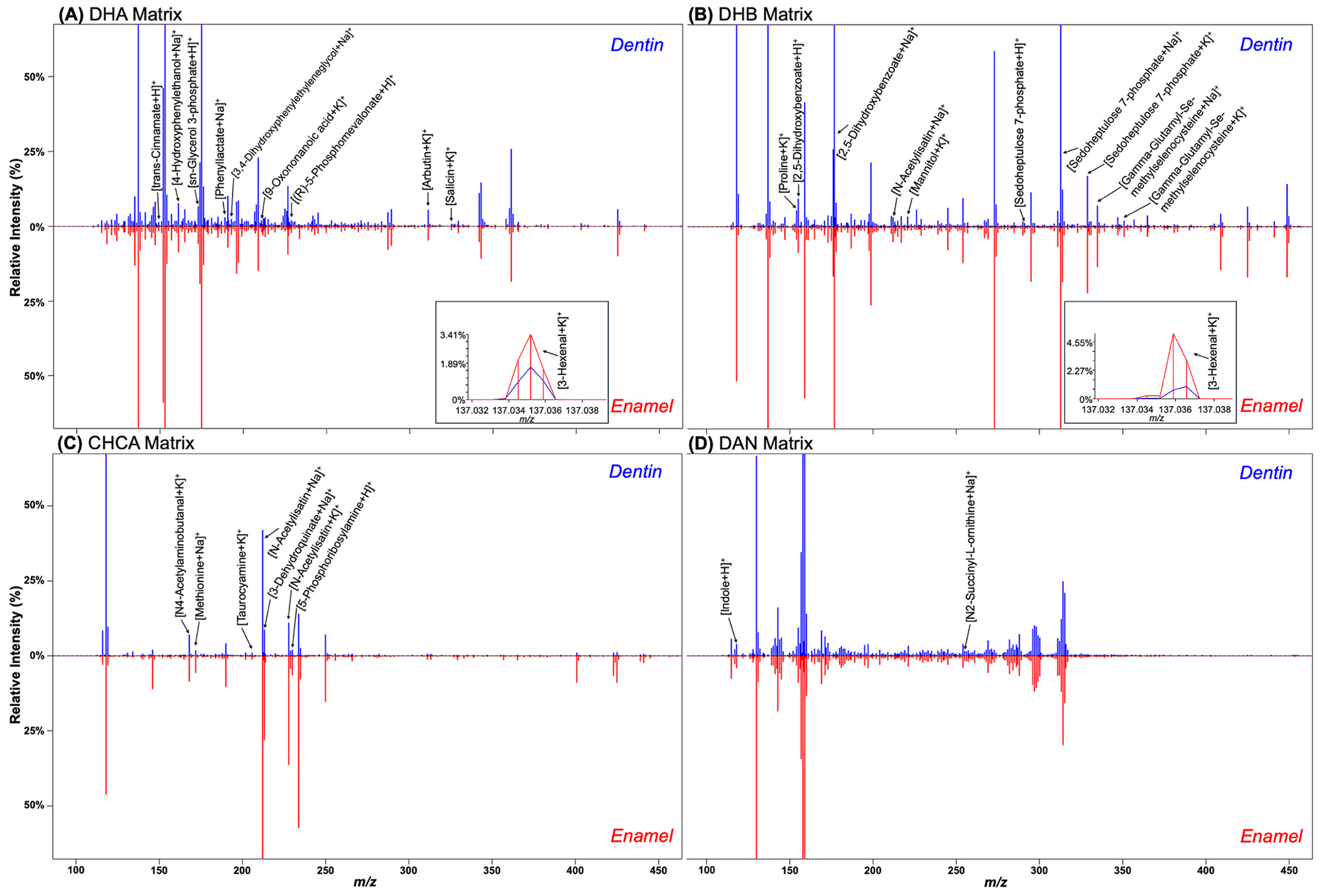
Various matrices resulted in different small molecule peaks observed in the non-decalcified human teeth. Differences in small molecules peak intensities were observed in dentin (top, blue) and enamel (bottom, red). The total number of features with an intensity of at least 1% of the base peak varies by matrix. There were 93 total features when using DHA (A), 59 total features using DHB (B), 31 total features using CHCA (C), and 74 total features using DAN (D). Some potential metabolites matched to the KEGG database via Pathos web facility are labeled for each mass spectrum (A-D). The full list of potential metabolites suggested by Pathos can be found in SI Table S3. Mass spectra scaled to 100% can be found in SI Figure S7. (For interpretation of the references to colour in this figure legend, the reader is referred to the Web version of this article.)

To evaluate matrix performance for small molecule detection in non-decalcified human teeth by MALDI mass spectrometry imaging (MSI), we conducted a comparative analysis of four matrices: DHA, DHB, CHCA, and DAN (Fig. 4). CHCA and DHB yielded the highest signal to noise ratios at the base peak (401 and 358 S/N, respectively) compared to DHA and DAN (225 and 153 S/N, respectively). DHA yielded the highest number of spectral features and potential metabolite annotations (93 features, 14 annotations, Fig. 4A–SI Figure S8), followed by DHB (59 features, 14 annotations, Fig. 4B), CHCA (31 features, 8 annotations, Fig. 4C), and DAN (74 features, 2 annotations, Fig. 4D). Most putative metabolite assignments are associated with amino acid metabolism, which is consistent with the protease function of saliva. Upon MS/MS analysis of Pathos annotated mass spectral features, inconclusive fragmentation patterns were observed. The Pathos annotations are highly putative and structural identifications were not determined for the purpose of this experiment. The presence of these small molecules in mineralized tissue sections highlights the potential of MALDI MSI for capturing biologically meaningful chemical signatures within the spatial architecture of human teeth.

Overall, these findings support the use of DHA as a matrix for small molecule analysis in non-decalcified teeth by MALDI MSI. Its performance in both feature count and metabolite annotation suggest potential for greater sensitivity and broader chemical coverage. Future studies could include further experiments to identify metabolites, enhancing the biological interpretation of metabolite distributions within dental microstructures. Although full metabolite identification remains unexplored for the purpose of this experiment, these results demonstrate that MALDI MSI can be applied to non-decalcified human teeth to yield spatially resolved chemical information. This method offers a foundation for future studies focused on developmental biology, environmental exposures, or disease biomarkers in mineralized tissues, provided that additional steps are taken to confirm compound identity and improve biological interpretation.

Napthalene and carbaryl insectide are two common environmental exposures, both of which result in the 1-naphthol metabolite in humans. Naphthalene is a polycyclic aromatic hydrocarbon (PAH), which tend to be bioaccumulating carcinogens [48]. PAHs are released into the air by incomplete combustion reactions, including forest fires, engine exhaust fumes, and are found in food such as cereal, vegetables, and meat cooked over an open flame [49]. Naphthalene is also used as a raw material for plasticizers, Similarly, carbaryl insecticide is also ubiquitous in the environment and is a widely used insecticide used on food crops, especially vegetables, under the brand name Sevin. It continues to be used on crops in the United States, but has been banned in certain countries and is classified as a likely carcinogen by the U.S. Environmental Protection Agency (EPA) [50]. The United States Center for Disease Control (CDC) released a report in 2003 measuring several chemicals in urine from the population, including 1-naphthol as a metabolite of napthalene and carbaryl insectide. In the report, 1-naphthol was measurable over 1 μg/L limit of detection in the majority of the sample population aged 6-59, without significant differences across age, gender, or ethnic groups [51].

**Fig. 5.**
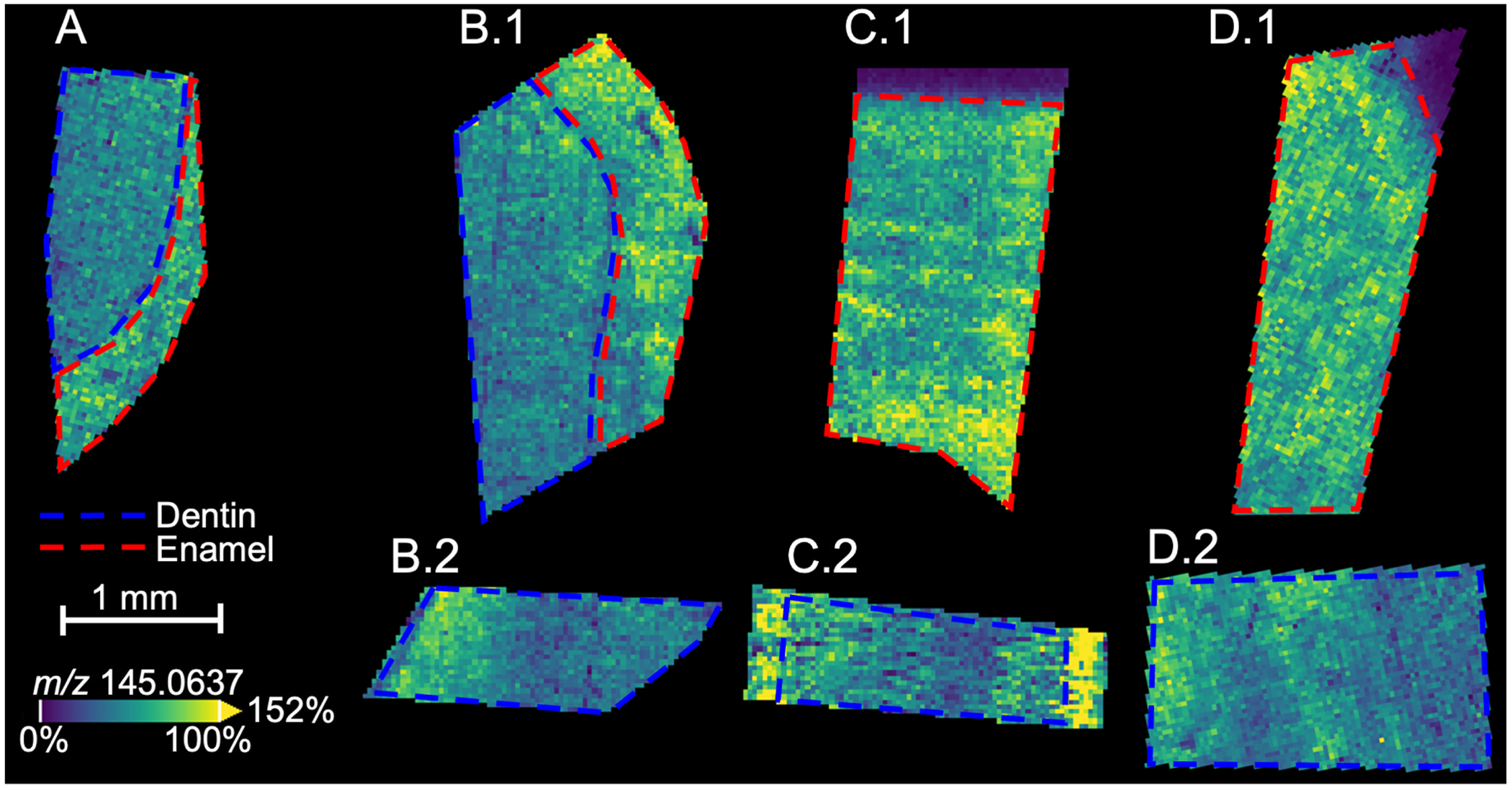
Spatial distribution of [1-naphthol) + H]^+^ (*m/z* 145.0637) found in non-decalcified human teeth analyzed by MALDI MSI with DHA matrix. This mass spectral feature was present in the crown of deciduous canine (A), crown of deciduous molar (B.1), root of deciduous molar (B.2), crown of permanent canine (C.1), root of permanent canine (C.2), crown of permanent molar (D.1), and root of permanent molar (D.2). MS/MS spectrum can be found in SI Figure S9.

In this experiment, the mass spectral feature *m/z* 145.0637 was consistent with [1-naphthol) + H]^+^, as confirmed by MS/MS fragmentation pattern matched to MassBank mass spectral library (SI Figure S9) [40]. Notably, the [1-naphthol) + H]^+^ (*m/z* 145.0637) mass spectral feature had a higher intensity in enamel than dentin in all four teeth (Fig. 5; box plots available in SI Figure S10). The deciduous incisor (Fig. 5A) and deciduous molar (Fig. 5B.1–2) each resulted in a [1-naphthol) + H]^+^ (*m/z* 145.0637) peak 1.3 times more intense in enamel than dentin. Similarly, the same mass spectral feature was 1.3–1.4 times more intense in enamel than dentin of permanent incisors (Fig. 5C.1–2) and permanent molars (Fig. 5D.1–2). The far right and left edges of the permanet incisor root (Fig. 5C.2) were discluded from the dentin region of the tooth due to likely representation of the cementum region of the tooth. Cementum is a mineralized layer covering on the root of the tooth, but contains less calcium and more organic matter compared to dentin [52]. To maintain enamel and dentin region exclusivity, the cementum was discluded from dentin regions and not considered further for the purpose of this experiment. In agreement with CDC findings, [1-naphthol) + H]^+^ (*m/z* 145.0637) is observed here in similar abundance across deciduous childhood teeth and permanent adult teeth samples. While this sample group is too small to make claims about the population, the heterogenous presence of [1-naphthol) + H]^+^ (*m/z* 145.0637) in the above non-decalcified, unfixed human teeth exemplifies a use case for this methodology.

## 5. Conclusion

This study outlines a method for preparing non-decalcified human teeth for MALDI mass spectrometry imaging (MSI), enabling spatially resolved chemical analysis of intact mineralized dental tissues. By using intact mineralized dental tissues, endogenous molecules are preserved by avoiding risks associated with chemical treatment for decalcification. Key technical components include the use of cryofilm to preserve spatial fidelity during section transfer, strong tungsten-carbide blades to minimize blade deformation and breakage and embedding in carboxymethyl cellulose (CMC) to provide structural support during cryosectioning. This method retains spatial distribution of molecular information found in the teeth, which could otherwise be lost during pulverization for bulk analysis. The spatial localization of the tentative lipid signals is encouraging, particularly at the dentin-pulp interface, where odontoblasts are known to form dentin during tooth development. Although lipid detection was sparse in the current dataset, these findings suggest the potential for the analysis of lipid species associated with odontoblast activity and dentinogenesis. With high spatial resolution, the thin dentin-enamel junction could be studied to target the organic matter within. Although microdissection has repeatedly achieved isolation of the pre-natal and post-natal tissue regions according to the neonatal line, sectioned teeth samples could increase the specificity and accessibility of region-specific studies. The primary limitation of this method is due to the crystalline nature of hydroxyapatite, which could be affecting the ability to ionize molecules in teeth by laser ablation. Additionally, this method could benefit by using a cryomicrotome that can be set up to minimize cryofilm bending during sectioning. The cryomicrotome used herein was adjusted within the limits of the instrument, but the cryofilm must bend during sectioning due to the non-parallel orientation of the tissue block and stage. Future work will focus on enhancing lipid extraction and ionization, with the goal of detecting free fatty acids or other blood-derived molecules incorporated into the tooth matrix during formation. Furthermore, this method could be adapted for detection of specific molecular classes in teeth such as peptides or small metabolites. Similar to MALDI MSI of soft tissue, analytes of interest could expand for various applications depending on the chosen matrix and MALDI MSI method. Even with this, there are limitations when considering many endogenous molecules are located crystal structure of hydroxyapatite and this method will not release many of these trapped molecules.

This method provides a foundation for future investigations into the spatial and temporal distribution of small molecules in human teeth. As dentin grows incrementally throughout a lifetime, it offers a unique time-resolved archive of biomolecules present in the blood during tooth development. While temporally resolved molecular patterns were not observed in the present study, such molecules are still expected to be preserved within this incremental structure and may be revealed in future work. By preserving this spatial and temporal structure, the method described here opens new possibilities for studying early-life exposures, developmental biology, and systemic disease markers. Applications may include the analysis of childhood diseases, localization of environmental exposure biomarkers, and the chemical analysis of archeological dental specimens. Specific biological questions such as molecular host-pathogen interactions at an infection site or the fundamental molecular makeup of the microscopic dentin-enamel junction could benefit from spatial analysis.

## Supplementary Material

1

## Data Availability

Data will be made available on request.

## HIGHLIGHTS

- Chemical Imaging of a variety of teeth samples (e.g. molar, canine, deciduous teeth).
- Teeth are not chemically altered, enabling direct molecular imaging.
- This technology can be used for future exposure experiments.

## Appendix A. Supplementary data

Supplementary data to this article can be found online at https://doi.org/10.1016/j.aca.2026.345386.
