# Supplementary material for: Non-decalcified human teeth sample preparation method for MALDI mass spectrometry imaging
Source: Anal Chim Acta. Author manuscript; Available in PMC 2026 Jun 11. (PMC13253108; doi:10.1016/j.aca.2026.345386)
Supplement: 1 [file NIHMS2180129-supplement-1.docx]

**Non-Decalcified Human Teeth Sample Preparation Method for MALDI Mass Spectrometry Imaging**

Kayle J. Bender^1^, Joanna E. Spurgeon^2^, Chuo Ying Zhai^1^, Manish Arora^2,3^, Elizabeth K. Neumann^1,*^

^1^Department of Chemistry, University of California, Davis, One Shields Avenue, Davis, California 95616, United States

^2^Linus Biotechnology, Inc., North Brunswick Township, NJ 08902, United States

^3^Icahn School of Medicine at Mount Sinai, New York, NY 10029, United States

**Supporting Information**

**Table of Contents**

| **SI Table S1:** Matrix Spray Parameters | Page 3 |
| --- | --- |
| **SI Figure S1:** MALDI MSI methods | Page 4 |
| **SI Figure S2:** Sample preparation method workflow | Page 5 |
| **SI Figure S3:** Embedding material effect on tooth section cracking | Page 6 |
| **SI Figure S4:** Blade angle effect on tooth section cracking | Page 7 |
| **SI Figure S5:** Ion images of lipid features (CHCA matrix) | Page 8 |
| **SI Figure S6:** Ion images of lipid features (DHB matrix) | Page 9 |
| **SI Figure S7:** Figure 4 scaled to 100% | Page 10 |
| **SI Figure S8:** Ion images of Pathos matched mass spectral features from DHA matrix experiment | Page 11 |
| **SI Figure S9:** On-tissue MS/MS fragmentation matching [1-naphthol)+H]^+^ | Page 12 |
| **SI Figure S10:** Region specific box plots of *m/z* 145.0637 | Page 13 |
| **SI Figure S11:** On-tissue MS/MS of [SM(34:1;O2)+H]^+^ | Page 14 |
| **SI Figure S12:** On-tissue MS/MS of [SM(34:1;O2)+Na]^+^ | Page 15 |
| **SI Table S2:** Potential peptides list | Page 16-18 |
| **SI Table S3:** Potential metabolites list | Page 19-20 |
| **References** | Page 21 |

Page 1

|  | **DHA** | **DHB** | **DAN** | **CHCA** |
| --- | --- | --- | --- | --- |
| **Solvent** | 70% acetonitrile | 70% methanol | tetrahydrofuran | 70% methanol |
| **Conc. (mg/mL)** | 10 | 40 | 20 | 5 |
| **Temp. (˚C)** | 50 | 75 | 40 | 80 |
| **Pressure (psi)** | 15 | 10 | 15 | 15 |
| **Flow Rate (µL/min)** | 120 | 100 | 50 | 120 |
| **Velocity (mm/min)** | 1200 | 1200 | 1350 | 1200 |
| **Track Spacing (mm)** | 2.5 | 3 | 2 | 2 |
| **Number of Passes** | 8 | 8 | 5 | 8 |
| **Pattern** | VV | CC | CC | CC |

**Table S1.** Matrix Spray Parameters

**Figure S1. MALDI MSI method details available for download as .zip file.**


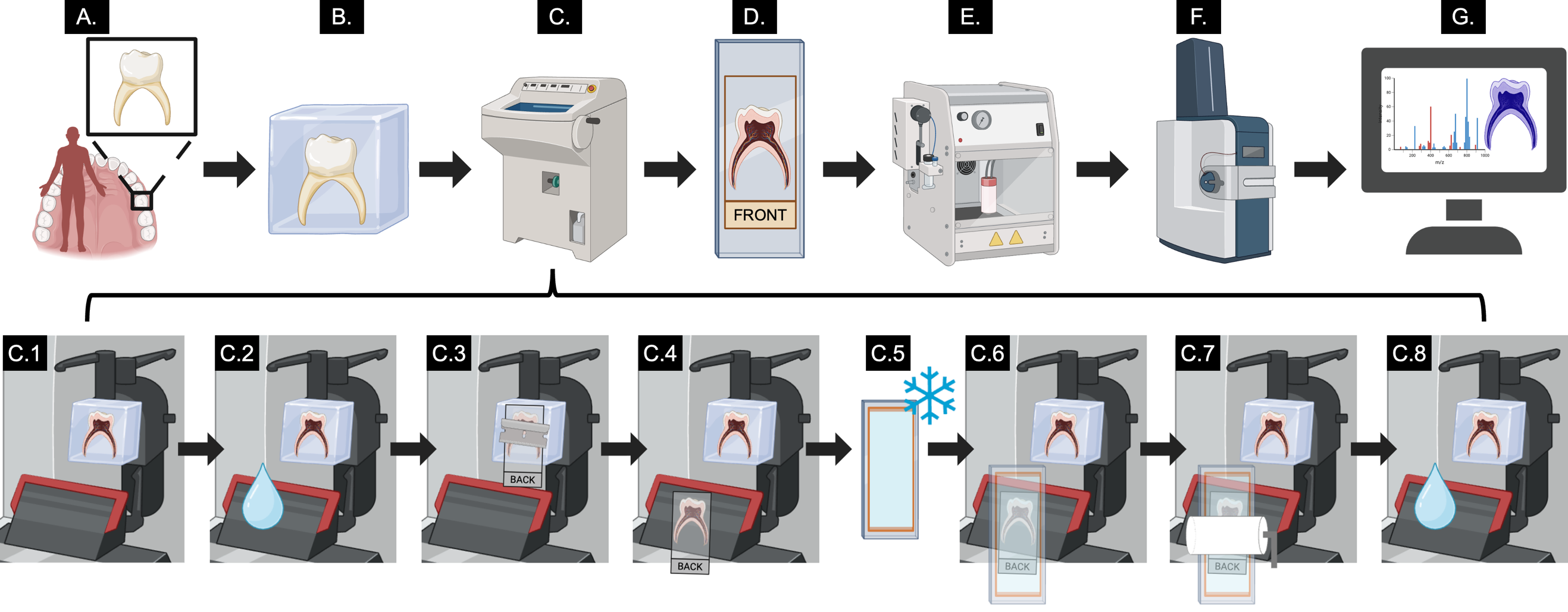


**SI Figure S2. Workflow to prepare non-decalcified, unfixed human teeth for MALDI MSI.** Collect samples (A), embed non-decalcified human teeth in CMC (B), section using cryomicrotome and cryofilm (C), heat-fix cryofilm on ITO-coated glass slide at 37˚C (D), spray coat matrix on slide (E), perform MALDI MSI using Bruker timsTOF fleX (F), then analyze MALDI MSI data (G). Cryomicrotome sectioning is further divided into several steps (C.1-8). First, the tissue block is mounted in the cryomicrotome and faced in 5-10 µm increments using SL series tungsten-carbide blades (C.1). Second, the cryomicrotome is thoroughly cleaned with methanol and a kimwipe prior to collecting the target tissue section (C.2). Third, the cryofilm is adhered to sample surface with non-adhesive color-coded edge closest to the stage and firmly adhered to the sample surface using a razor blade (C.3). Note, the color-coded edge is added by the manufacturer to visually differentiate the adhesive side of the cryofilm (yellow, “front”) from the non-adhesive side (grey, “back”) and provide a non-adhesive edge. Fourth, the target tissue section is sectioned from the tissue block, landing on the cryomicrotome stage surface (C.4). Prepare ITO-coated glass slide by applying copper tape, then ZIG 2-way glue, drying until clear, and cooling in cryomicrotome (C.5). With tooth section face down on stage, press glue side of slide onto the non-adhesive side of the cryofilm (C.6). If necessary, use forceps to pick up and move the cryofilm to another clean part of the stage without dragging the cryofilm across the stage. To eliminate air bubble between the cryofilm and glue, use a chilled PTFE roller to press the slide firmly against the cryofilm on the stage (C.7). Always clean the stage thoroughly with methanol and a kimwipe between each cryofilm section (C.8). Figure created with BioRender.com

**
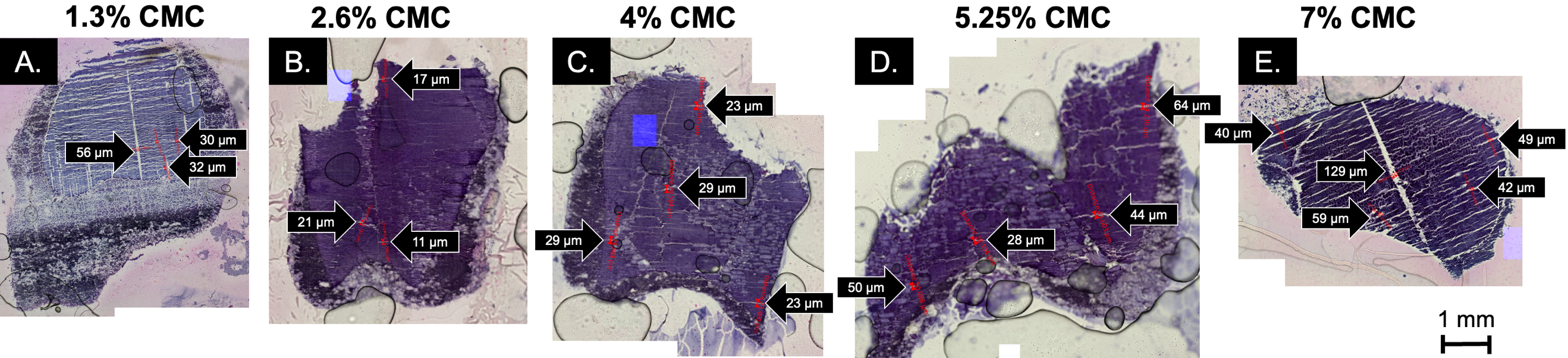
**

**SI Figure S3: Embedding material effect on tooth section cracking.** Teeth embedded in 1.3%, 2.6%, 4%, 5.25%, or 7% carboxymethylcellulose (CMC) were sectioned and hematoxylin and eosin (H&E) stained (A-E, respectively). Several of the widest cracks in each tooth section were measured via microscopy annotation (red text). Cracks measurement areas are highlighted by black arrows, displaying the measurement in micrometers rounded to the nearest whole number.

**
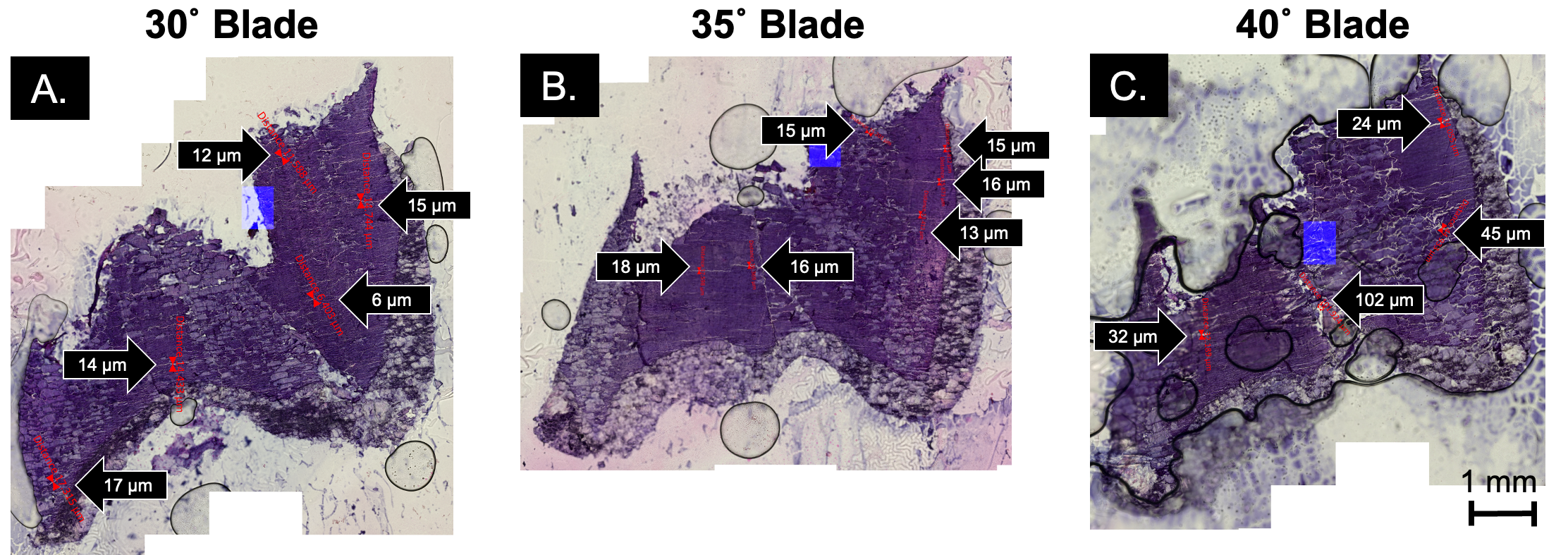
**

**SI Figure S4: Blade angle effect on tooth section cracking.** Representative hematoxylin and eosin (H&E) stained sections of non-decalcified human teeth were prepared using 30, 35, and 40 degree SL series tungsten-carbide blades (A-C, respectively). Several of the widest cracks in each tooth section were measured via microscopy annotation (red text). Cracks measurement areas are highlighted by black arrows, displaying the measurement in micrometers rounded to the nearest whole number.


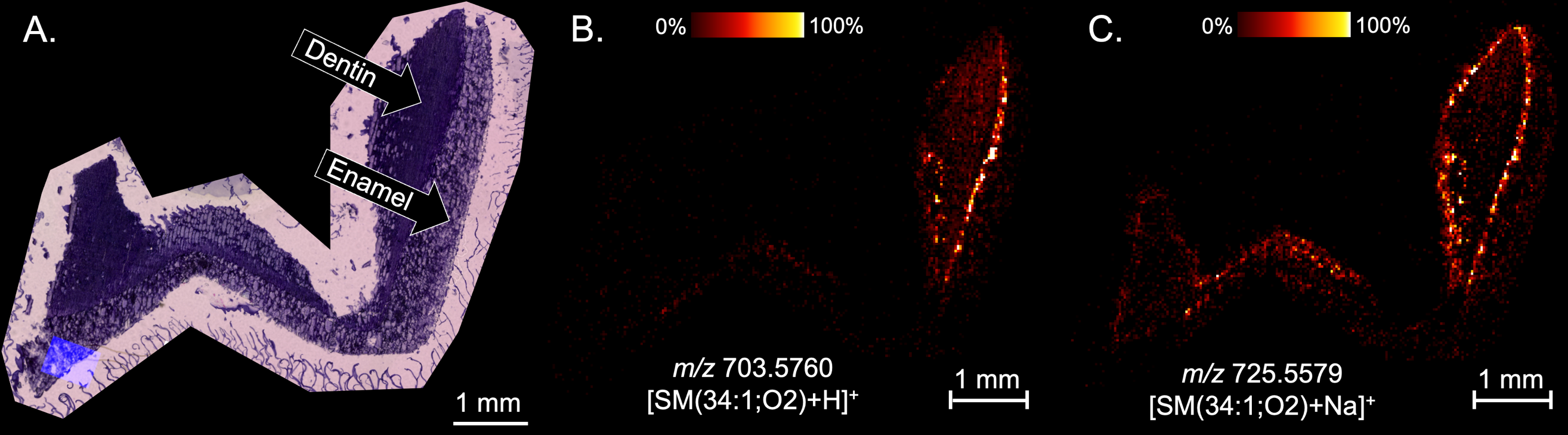


**SI Figure S5. Ion images of mass spectral features consistent with lipids using CHCA matrix.** Non-decalcified human teeth thin sections consist of both dental and enamel (A). MALDI MSI of non-decalcified human teeth using CHCA matrix yielded two mass spectral features consistent with lipids were annotated as [SM(34:1;O2)+H]^+^ (B) and [SM(34:1;O2)+Na]^+^ (C). On-tissue tandem MS of each isolated peak revealed the diagnostic phosphocholine headgroup at *m/z* 184. Both lipid features are primarily localized to the dentin-enamel junction or at the interface between dentin and pulp in this tooth.


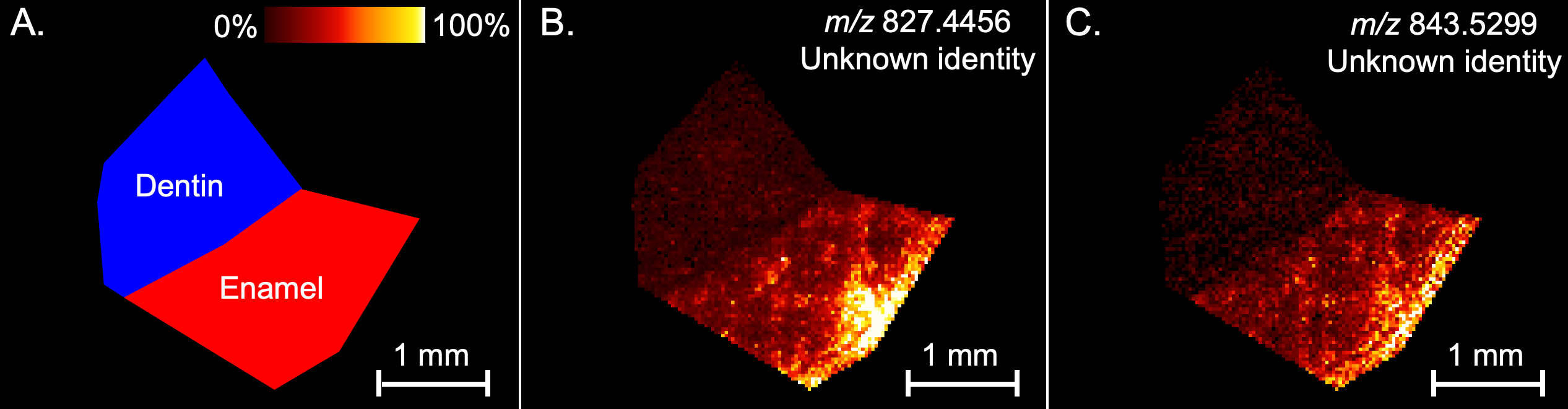


**SI Figure S6. Ion images of mass spectral features consistent with lipids using DHB matrix.** Dental and enamel and both present in non-decalcified human teeth thin sections (A). MALDI MSI of non-decalcified human teeth DHB matrix resulted in two mass spectral features consistent with lipids. Neither lipid feature was identified by *m/z* value (B-C). Both lipid features are primarily localized to the enamel in this tooth.


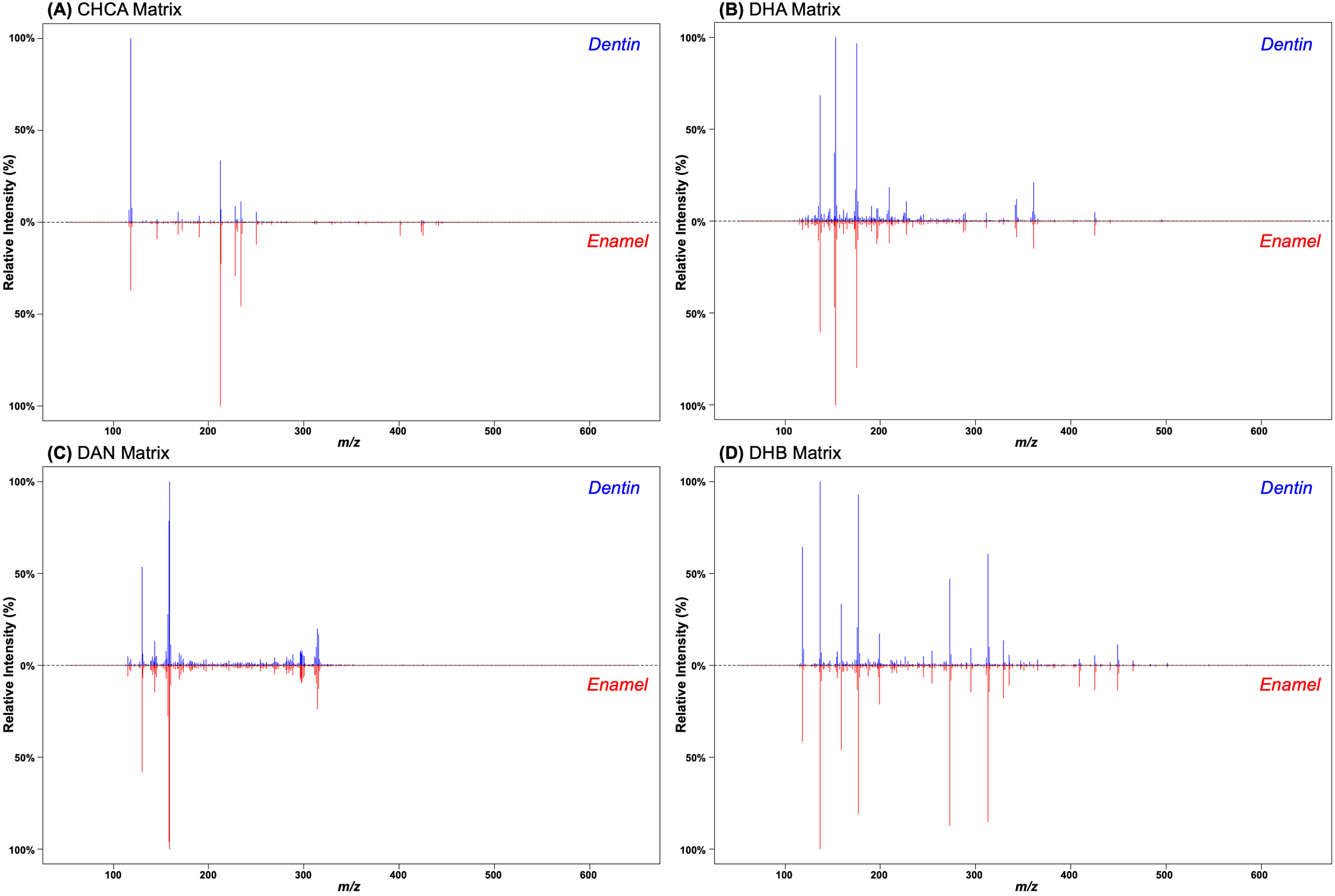


**SI Figure S7. Full mass spectra for data shown in Figure 4.** Small molecule detection in non-decalcified, unfixed human teeth was explored using CHCA (A), DHA (B), DAN (C), and DHB (D) matrices. Differences in small molecules peak intensities were observed in dentin (top, blue) and enamel (bottom, red).


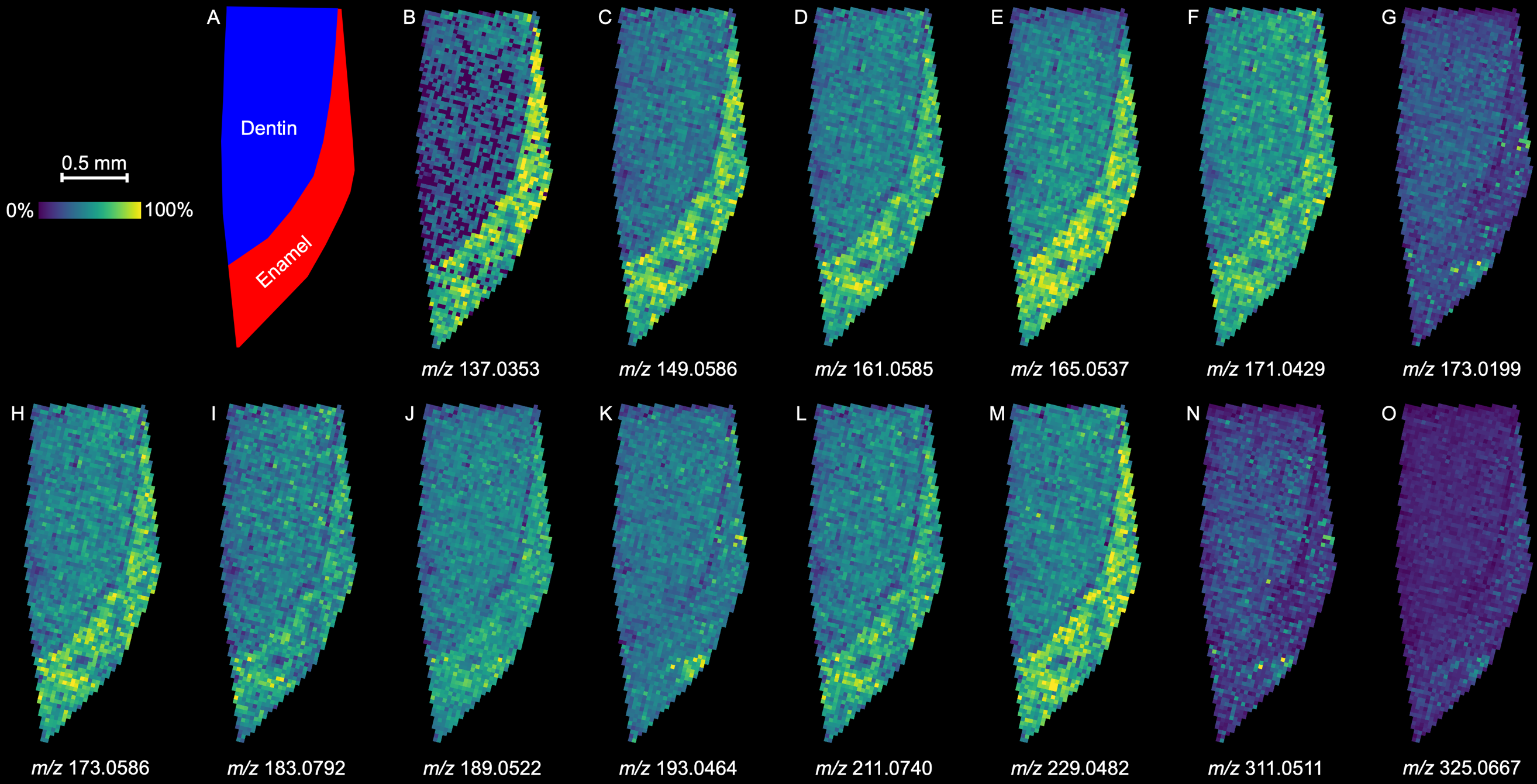


**SI Figure S8. Spatial distribution of potential metabolites found in non-decalcified human teeth analyzed by MALDI MSI with DHA matrix.** The tooth is sectioned vertically, with dentin and enamel regions present (A). There were 14 potential metabolite peaks found in this experiment (B-O). Each potential metabolite peak is represented above by an ion image heatmap, an *m/z* value, and a putative metabolite annotation. The full list of potential metabolites found using KEGG database via Pathos web facility is included in SI Table S3.

**SI Figure S9. On-tissue MS/MS of *m/z* 145.0656.** Collision induced dissociation (collision energy of 20 eV) was used to fragment *m/z* 145.0656, resulting in two fragments (*m/z* 115.0541 and *m/z* 117.0708) associated with 1-naphthol according to MassBank (accession IDs: MSBNK-LCSB-LU113601 and MSBNK-LCSB-LU113602).^1^


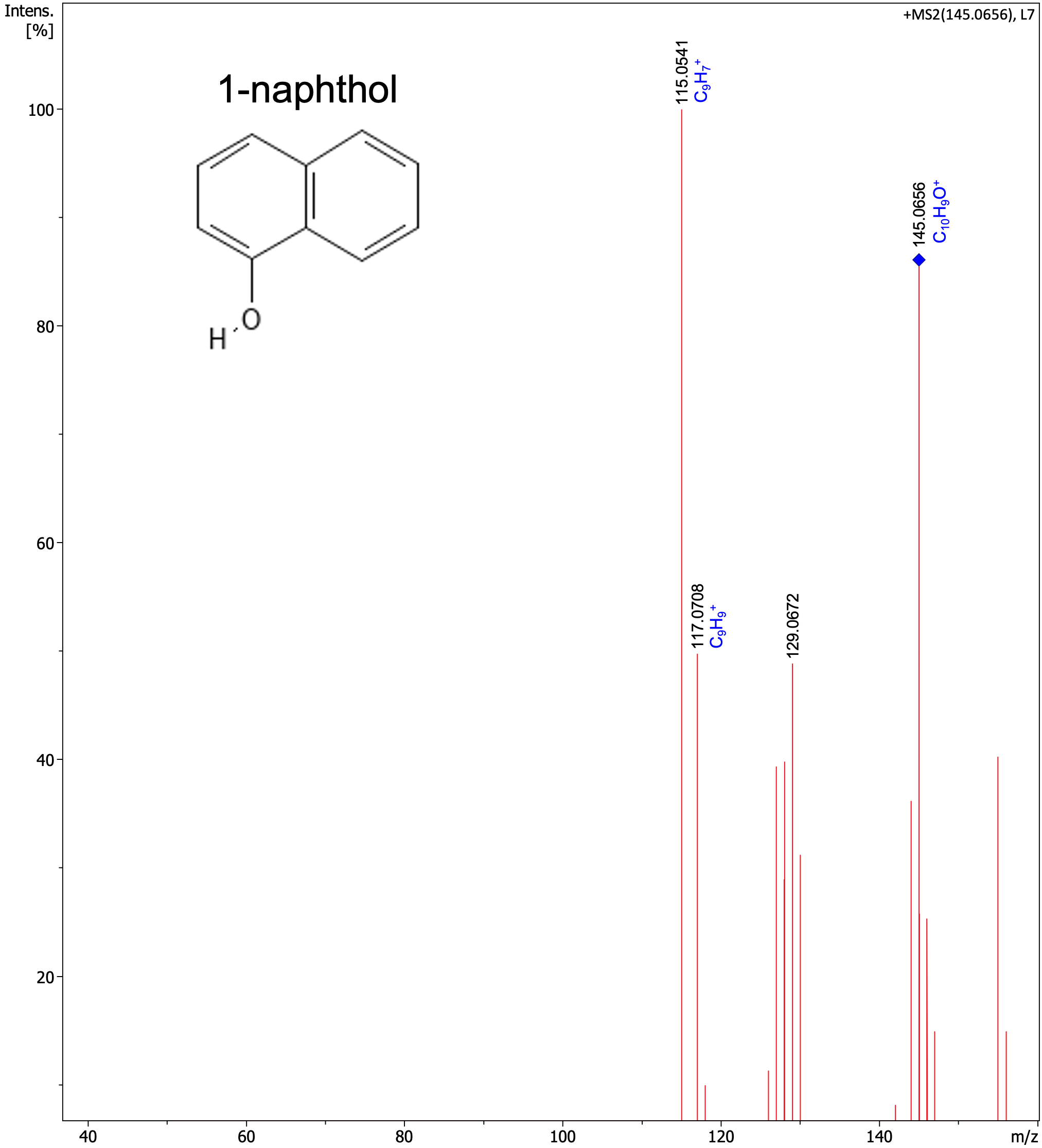


**
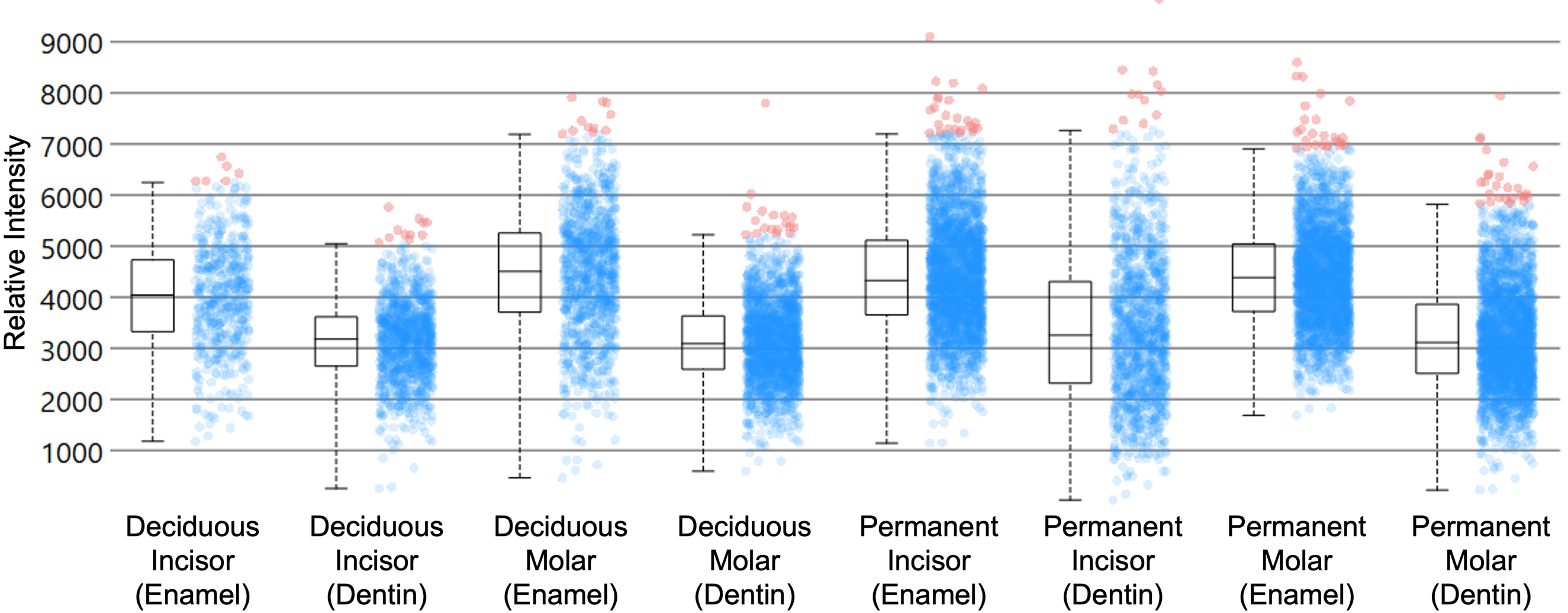
**

**SI Figure S10:** **Intensity box plot comparison of mass spectral feature *m/z* 145.0637 in dentin and enamel regions of non-decalcified, unfixed human teeth.** Box plots from SCiLS Lab software compares the intensity of *m/*z 145.0637 in the enamel and dentin regions of deciduous incisors, deciduous molars, permanent incisors, and permanent molars, as labeled. Dots represent the intensity of *m/*z 145.0637 for each MALDI MSI ion image pixel.

**
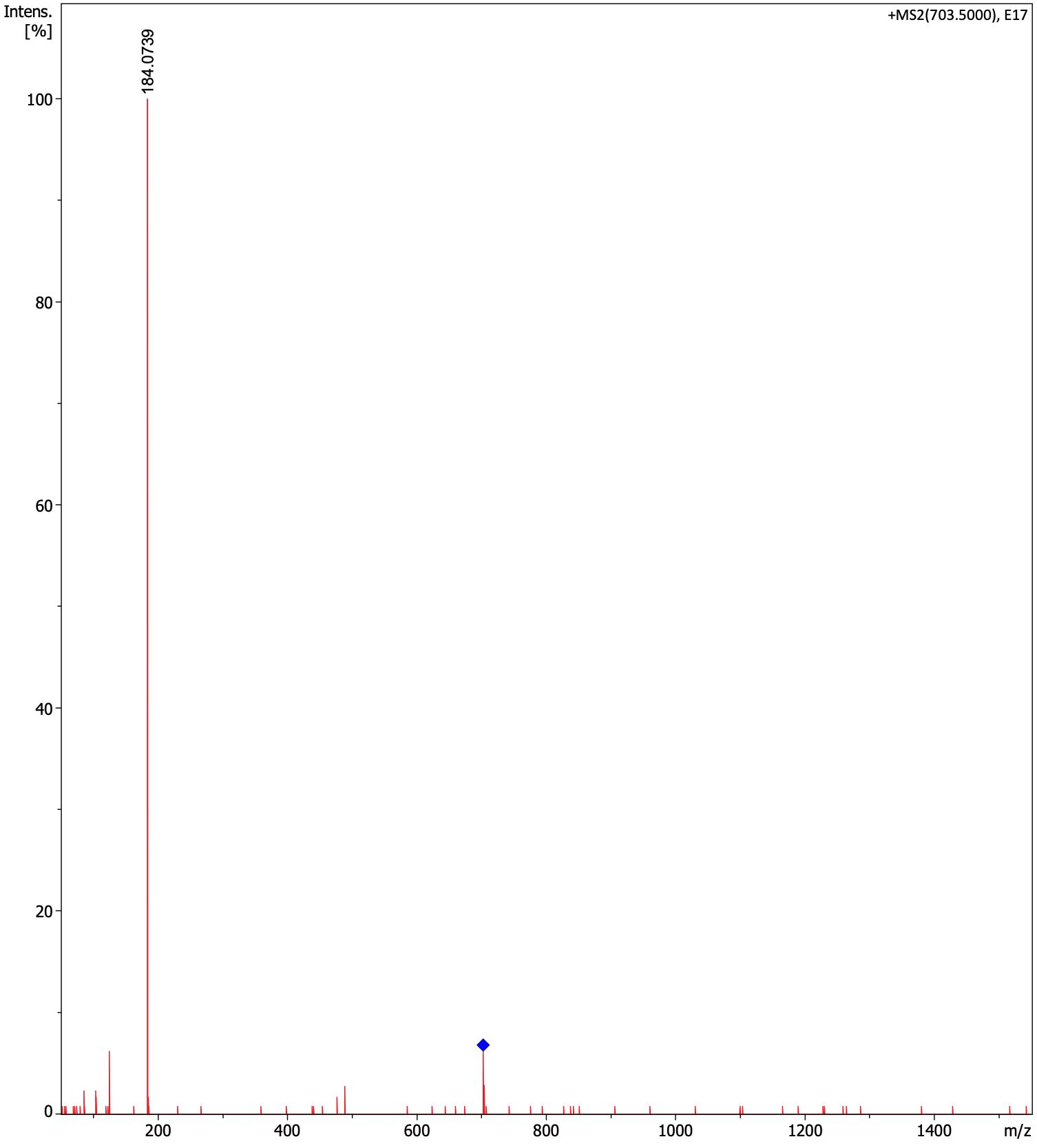
**

**SI Figure S11:** MS/MS of *m/z* 703.5, annotated as [SM(34:1;O2)+H]^+^. The peak at *m/z* 184 corresponds to the loss of the phosphocholine head group [C_5_H_14_O_4_PN+H]^+^.

**
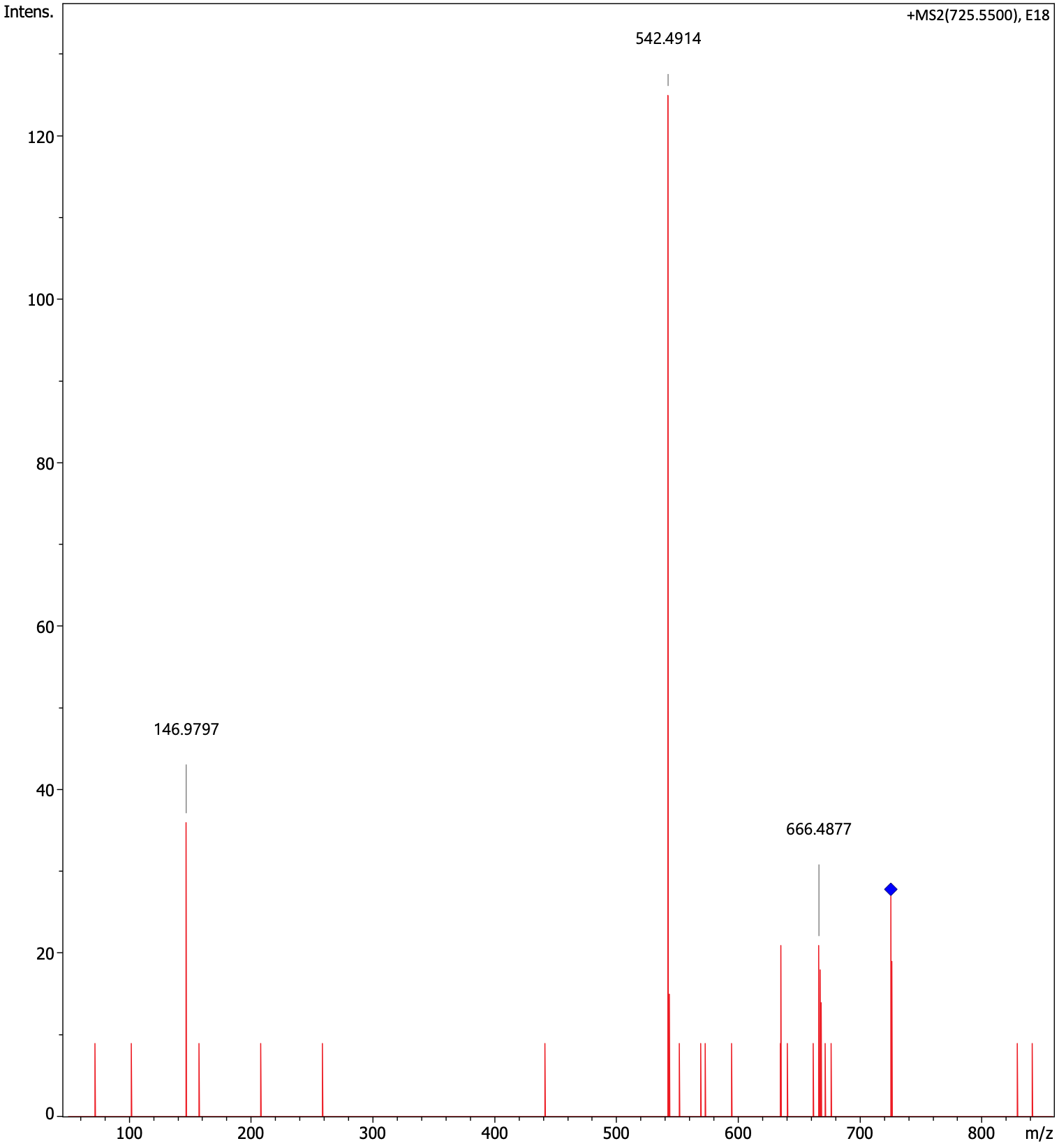
**

**SI Figure S12:** MS/MS of *m/z* 725.5, annotated as [SM(34:1;O2)+Na]^+^. The peak at *m/z* 542 corresponds to the loss of the phosphocholine head group, -183 Da from the parent ion.

**Table S2.** Potential peptide list via METLIN^2^

| **DHA Matrix** | | | | |
| --- | --- | --- | --- | --- |
|  | **METLIN Database Match** | | | |
| *m/z* | Chemical Formula | Adduct | Potential Peptide | ppm error |
| 441.1833 | C16H24N8O7 | [M+H]⁺ | Asn His Asn Gly | 3.00 |
| 442.184 | C15H29N7O5S | [M+Na]⁺ | Met Gly Arg Gly | 1.97 |
| 457.2659 | C21H36N4O7 | [M+H]⁺ | Val Pro Leu Glu | 0.71 |
| 478.0882 | C14H25N5O6S3 | [M+Na]⁺ | Gln Cys Cys Cys | -3.62 |
| 479.1843 | C18H28N6O8 | [M+Na]⁺ | Val Ser His Asp | 4.95 |
| 593.2202 | C28H34N4O9 | [M+Na]⁺ | Tyr Tyr Pro Glu | 3.66 |
| 595.2297 | C32H36N4O5 | [M+K]⁺ | Pro Phe Phe Phe | 4.38 |
| 599.2353 | C25H36N8O7 | [M+K]⁺ | Tyr Asn Lys His | -1.49 |
| 639.2554 | C32H36N6O7 | [M+Na]⁺ | Glu Trp Pro Trp | -1.77 |
| **DHB Matrix** | | | | |
|  | **METLIN Database Match** | | | |
| *m/z* | Chemical Formula | Adduct | Potential Peptide | ppm error |
| 403.2355 | C21H30N4O4 | [M+H]⁺ | Trp Val Val | -2.42 |
| 425.2176 | C18H34N4O5 | [M+K]⁺ | Leu Ala Leu Ala | -2.33 |
| 441.1917 | C18H28N6O5S | [M+H]⁺ | Pro Met His Gly | 0.74 |
| 479.1937 | C17H30N6O8S | [M+H]⁺ | Gly Thr Gln Cys Ala | -2.66 |
| 517.0804 | C16H22N4O13 | [M+K]⁺ | Asp Asp Asp Asp | 3.11 |
| **CHCA Matrix** | | | | |
|  | **METLIN Database Match** | | | |
| *m/z* | Chemical Formula | Adduct | Potential Peptide | ppm error |
| 329.1488 | C18H20N2O4 | [M+H]⁺ | Tyr Phe | 4.03 |
| 425.2163 | C21H30N4O4 | [M+Na]⁺ | Trp Val Val | 0.40 |
| 441.1907 | C18H28N6O5S | [M+H]⁺ | Pro Met His Gly | 3.00 |
| 636.243 | C33H35N5O7 | [M+Na]⁺ | Trp Phe Phe Asp | 0.74 |
| **DAN Matrix** | | | | |
|  | **METLIN Database Match** | | | |
| *m/z* | Chemical Formula | Adduct | Potential Peptide | ppm error |
| 403.2333 | C21H30N4O4 | [M+H]⁺ | Trp Val Val | 3.04 |
| 414.1776 | C15H29N5O5S | [M+Na]⁺ | Met Lys Gly Gly | 2.58 |
| 425.2151 | C18H28N6O6 | [M+H]⁺ | Thr Pro His Ala | -0.65 |
| 427.1407 | C19H24N4O4S1 | [M+Na]⁺ | Pro Cys Trp | 2.04 |
| 428.1442 | C17H23N7O4 | [M+K]+ | His His Pro | 1.65 |
| 431.2072 | C20H32N4O4 | [M+K]⁺ | Phe Val Lys | -2.53 |
| 437.119 | C15H22N6O7 | [M+K]⁺ | His Gly Gly Glu | -0.67 |
| 441.1902 | C18H28N6O5S | [M+H]⁺ | Pro Met His Gly | 4.14 |
| 445.2212 | C21H34N4O4 | [M+K]⁺ | Phe Lys Leu | 1.14 |
| 459.2311 | C20H32N6O5 | [M+Na]⁺ | Pro Leu His Ala | 4.51 |
| 471.1656 | C18H26N6O7S | [M+H]⁺ | Pro His Asp Cys | 1.33 |
| 479.206 | C19H32N6O5 | [M+Na]⁺ | Val Val His Cys | -1.52 |
| 490.2039 | C21H27N7O7 | [M+H]⁺ | Tyr Asn His Gly | 2.29 |
| 492.2184 | C19H31N7O7 | [M+Na]⁺ | Val Thr Asn His | -0.26 |
| 498.1291 | C22H25N3O8 | [M+K]⁺ | Tyr Asp Tyr | -2.39 |
| 500.2886 | C26H37N5O5 | [M+H]⁺ | Trp Val Val Pro | -2.55 |
| 502.3015 | C26H39N5O5 | [M+H]⁺ | Trp Val Val Val | 2.84 |
| 507.2235 | C19H34N6O8S | [M+H]⁺ | Thr Gln Gln Met | 0.44 |
| 509.1796 | C24H30N4O6 | [M+K]⁺ | Tyr Phe Ala Ala | 1.19 |
| 516.2175 | C21H31N7O7 | [M+Na]⁺ | Arg Gly Phe Asp | 1.49 |
| 520.1828 | C21H31N5O7S | [M+Na]⁺ | Tyr Val Asn Cys | 2.63 |
| 526.2056 | C19H33N7O7S | [M+Na]⁺ | Arg Pro Glu Cys | 0.70 |
| 533.1785 | C21H30N6O7S | [M+Na]⁺ | Gln Asn Phe Cys | 1.82 |
| 536.194 | C22H35N5O6S | [M+K]⁺ | Tyr Met Lys Gly | 0.95 |
| 544.1803 | C23H31N5O8 | [M+K]⁺ | Trp Val Ser Asp | 1.30 |
| 547.1927 | C22H32N6O8 | [M+K]⁺ | Tyr Val Asn Asn | -1.45 |
| 549.1458 | C19H34N4O8S2 | [M+K]⁺ | Thr Met Met Glu | -0.53 |
| 550.2087 | C23H31N7O7S | [M+H]⁺ | Tyr Gln His Cys | -0.50 |
| 561.3177 | C23H44N8O6S | [M+H]⁺ | Met Leu Arg Ala Ala | 1.11 |
| 566.1699 | C22H33N5O7S2 | [M+Na]⁺ | Tyr Gln Met Cys | 3.48 |
| 571.1944 | C24H32N6O7S | [M+Na]⁺ | Met His Phe Asp | 1.17 |
| 571.26 | C24H36N8O7 | [M+Na]⁺ | Tyr Arg Pro Asn | 0.82 |
| 576.2193 | C26H31N7O7 | [M+Na]⁺ | Trp Pro His Asp | -1.79 |
| 579.2302 | C25H32N8O7 | [M+Na]⁺ | Tyr Thr His His | -1.78 |
| 580.1811 | C26H31N5O8 | [M+K]⁺ | Tyr Trp Ser Ser | -0.16 |
| 581.1873 | C24H30N8O7 | [M+K]⁺ | Tyr Ser His His | 0.19 |
| 582.1901 | C25H33N7O5S | [M+K]⁺ | Trp Val His Cys | 0.01 |
| 583.1299 | C22H32N4O8S2 | [M+K]⁺ | Tyr Met Glu Cys | 0.01 |
| 583.1933 | C25H32N6O8 | [M+K]⁺ | Tyr Pro His Glu | -2.39 |
| 583.2048 | C29H32N6O5 | [M+K]⁺ | Trp Trp Pro Gly | 3.96 |
| 583.259 | C25H36N8O7 | [M+Na]⁺ | Tyr Asn Lys His | 2.52 |
| 587.1566 | C25H32N4O8S | [M+K]⁺ | Tyr Tyr Thr Cys | 2.06 |
| 588.217 | C24H35N7O8 | [M+K]⁺ | Tyr Arg Pro Asp | 2.39 |
| 591.1822 | C23H32N6O10 | [M+K]⁺ | Asn Asp Phe Ala Ser | -0.83 |
| 592.1837 | C27H31N5O7S | [M+Na]⁺ | Trp Phe Asp Cys | 0.79 |
| 593.1802 | C23H34N6O8S | [M+K]⁺ | Tyr Gln Asn Met | -1.00 |
| 594.1946 | C27H33N5O8 | [M+K]⁺ | Tyr Tyr Pro Asn | 3.38 |
| 595.1984 | C28H36N4O6S | [M+K]⁺ | Tyr Pro Met Phe | 1.53 |
| 597.2696 | C26H40N6O8S | [M+H]⁺ | Met Asn Ser Phe Val | 1.72 |
| 598.149 | C24H29N7O7S | [M+K]⁺ | Trp His Asp Cys | -0.66 |
| 598.2732 | C27H35N9O7 | [M+H]⁺ | His His Ala Ala Tyr | 0.88 |
| 601.2231 | C22H38N6O11 | [M+K]⁺ | Glu Glu Lys Ser Ala | 0.84 |
| 603.2292 | C28H34N4O11 | [M+H]⁺ | Tyr Tyr Glu Glu | 1.70 |
| 606.1964 | C28H33N5O8 | [M+K]⁺ | Tyr Trp Glu Ala | 0.34 |
| 608.2061 | C22H33N5O15 | [M+H]⁺ | Asp Ser Glu Glu Glu | -1.60 |
| 609.211 | C29H32N6O7S | [M+H]⁺ | Trp Trp Asp Cys | 3.49 |
| 612.221 | C31H35N5O6 | [M+K]⁺ | Asn Phe Phe Phe | 2.30 |
| 613.1704 | C27H34N4O8S | [M+K]⁺ | Tyr Met Phe Asp | 4.91 |
| 617.1861 | C30H34N4O6S | [M+K]⁺ | Tyr Phe Phe Cys | -4.04 |
| 620.2612 | C29H39N7O6 | [M+K]⁺ | Tyr Arg Pro Phe | -2.08 |
| 626.2343 | C30H33N7O7 | [M+Na]⁺ | Trp His Phe Asp | -0.69 |
| 633.2693 | C27H42N6O8S | [M+Na]⁺ | Ile Met Asn Ser Phe | -1.63 |

**Table S3.** Potential metabolites list via Pathos web facility^3^

| **DHA Matrix** | | | |
| --- | --- | --- | --- |
|  | **Pathos match to KEGG** | | |
| *m/z* | Chemical Formula | Potential Metabolite | Pathway associated with potential metabolite |
| 137.0353 | C6H10O(+K⁺) | [3-Hexenal+K]⁺ | alpha-Linolenic acid metabolism |
| 149.0586 | C9H8O2(+H⁺) | [trans-Cinnamate+H]⁺ | Phenylalanine metabolism |
| 161.0585 | C8H10O2(+Na⁺) | [4-Hydroxyphenylethanol+Na]⁺ | Tyrosine metabolism |
| 165.0537 | C9H8O3(+H⁺) | [Phenylpyruvate+H]⁺ | Phenylalanine metabolism |
|  |  | [enol-Phenylpyruvate+H]⁺ | Phenylalanine metabolism |
|  |  | [trans-2-Hydroxycinnamate+H]⁺ | Phenylalanine metabolism |
|  |  | [trans-3-Hydroxycinnamate+H]⁺ | Phenylalanine metabolism |
|  |  | [4-Coumarate+H]⁺ | Tyrosine metabolism |
| 171.0429 | C9H8O2(+Na⁺) | [trans-Cinnamate+Na]⁺ | Phenylalanine metabolism |
| 173.0199 | C3H9O6P(+H⁺) | [sn-Glycerol 3-phosphate+H]⁺ | Glycerophospholipid metabolism |
|  | C8H6O3(+Na⁺) | [alpha-Oxo-benzeneacetic acid+Na]⁺ | Phenylalanine metabolism |
|  | C5H10O4(+K⁺) | [deoxyribose+K]⁺ | Pentose phosphate pathway |
|  |  | [(R)-2,3-Dihydroxy-3-methylbutanoate+K]⁺ | Valine, leucine and isoleucine biosynthesis |
| 173.0586 | C9H10O2(+Na⁺) | [Phenylpropanoate+Na]⁺ | Phenylalanine metabolism |
| 183.0792 | C8H16O2(+K⁺) | [Octanoic acid+K]⁺ | Fatty acid biosynthesis |
| 189.0522 | C9H10O3(+Na⁺) | [Phenyllactate+Na]⁺ | Phenylalanine metabolism |
|  |  | [3-(2-Hydroxyphenyl)propanoate+Na]⁺ | Phenylalanine metabolism |
|  |  | [3-(3-Hydroxy-phenyl)-propanoic acid+Na]⁺ | Phenylalanine metabolism |
|  |  | [3-Methoxy-4-hydroxyphenylacetaldehyde+Na]⁺ | Tyrosine metabolism |
| 193.0464 | C8H10O4(+Na⁺) | [3,4-Dihydroxyphenylethyleneglycol+Na]⁺ | Tyrosine metabolism |
| 211.074 | C9H16O3(+K⁺) | [9-Oxononanoic acid+K]⁺ | alpha-Linolenic acid metabolism |
| 229.0482 | C6H13O7P(+H⁺) | [(R)-5-Phosphomevalonate+H]⁺ | Terpenoid backbone biosynthesis |
|  | C8H14O5(+K⁺) | [(R)-3-((R)-3-Hydroxybutanoyloxy)butanoate+K]⁺ | Butanoate metabolism |
| 311.0511 | C12H16O7(+K⁺) | [Arbutin+K]⁺ | Glycolysis / Gluconeogenesis |
| 325.0667 | C13H18O7(+K⁺) | [Salicin+K]⁺ | Glycolysis / Gluconeogenesis |
| **DHB Matrix** | | | |
|  | **Pathos match to KEGG** | | |
| *m/z* | Chemical Formula | Potential Metabolite | Pathway associated with potential metabolite |
| 137.0363 | C6H10O(+K⁺) | [3-Hexenal+K]⁺ | alpha-Linolenic acid metabolism |
| 154.0001 | C3H7NO2S2(+H⁺) | [Thiocysteine+H]⁺ | Cysteine and methionine metabolism |
| 154.0258 | C5H9NO2(+K⁺) | [Proline+K]⁺ | Arginine and proline metabolism |
| 155.0338 | C7H6O4(+H⁺) | [2,5-Dihydroxybenzoate+H]⁺ | Tyrosine metabolism |
| 159.0196 | C8H8O(+K⁺) | [Phenylacetaldehyde+K]⁺ | Phenylalanine metabolism |
| 177.0159 | C7H6O4(+Na⁺) | [2,5-Dihydroxybenzoate+Na]⁺ | Tyrosine metabolism |
| 177.0305 | C8H10O2(+K⁺) | [4-Hydroxyphenylethanol+K]⁺ | Tyrosine metabolism |
| 212.0321 | C10H7NO3(+Na⁺) | [N-Acetylisatin+Na]⁺ | Tryptophan metabolism |
|  |  | [4-Hydroxy-2-quinolinecarboxylic acid+Na]⁺ | Tryptophan metabolism |
|  | C7H11NO4(+K⁺) | [N-Acetyl-L-glutamate 5-semialdehyde+K]⁺ | Arginine biosynthesis |
| 221.0425 | C9H10O5(+Na⁺) | [3-Methoxy-4-hydroxymandelate+Na]⁺ | 3-Methoxy-4-hydroxymandelate |
|  | C6H14O6(+K⁺) | [Mannitol+K]⁺ | Fructose and mannose metabolism |
|  |  | [D-Sorbitol+K]⁺ | Galactose metabolism |
|  |  | [Galactitol+K]⁺ | Galactose metabolism |
| 291.0502 | C7H15O10P(+H⁺) | [Sedoheptulose 7-phosphate+H]⁺ | Pentose phosphate pathway |
|  | C10H12N4O4(+K⁺) | [Deoxyinosine+K]⁺ | Purine metabolism |
| 313.0324 | C9H16N2O5Se(+H⁺) | [Gamma-Glutamyl-Se-methylselenocysteine+H]⁺ | Selenoamino acid metabolism |
|  | C7H15O10P(+Na⁺) | [Sedoheptulose 7-phosphate+Na]⁺ | Pentose phosphate pathway |
| 329.0064 | C7H15O10P(+K⁺) | [Sedoheptulose 7-phosphate+K]⁺ | Pentose phosphate pathway |
| 335.0145 | C9H16N2O5Se(+Na⁺) | [Gamma-Glutamyl-Se-methylselenocysteine+Na]⁺ | Selenoamino acid metabolism |
| 350.9887 | C9H16N2O5Se(+K⁺) | [Gamma-Glutamyl-Se-methylselenocysteine+K]⁺ | Selenoamino acid metabolism |
| **DAN Matrix** | | | |
|  | **Pathos match to KEGG** | | |
| *m/z* | Chemical Formula | Potential Metabolite | Pathway associated with potential metabolite |
| 118.0654 | C8H7N(+H⁺) | [Indole+H]⁺ | Phenylalanine, tyrosine and tryptophan biosynthesis |
|  |  | [Phenylacetonitrile+H]⁺ | Cyanoamino acid metabolism |
| 255.0944 | C9H16N2O5(+Na⁺) | [N2-Succinyl-L-ornithine+Na]⁺ | Arginine and proline metabolism |
|  |  | [gamma-Glutamyl-gamma-aminobutyrate+Na]⁺ | Arginine and proline metabolism |
|  |  | [N6-Acetyl-LL-2,6-diaminoheptanedioate+Na]⁺ | Lysine biosynthesis |
| **CHCA Matrix** | | | |
|  | **Pathos match to KEGG** | | |
| *m/z* | Chemical Formula | Potential Metabolite | Pathway associated with potential metabolite |
| 168.0418 | C6H11NO2(+K⁺) | [N4-Acetylaminobutanal+K]⁺ | Arginine and proline metabolism |
|  |  | [L-Pipecolate+K]⁺ | Lysine degradation |
| 172.0392 | C5H11NO2S(+Na⁺) | [Methionine+Na]⁺ | Aminoacyl-tRNA biosynthesis |
| 190.0492 | C10H7NO3(+H⁺) | [N-Acetylisatin+H]⁺ | Tryptophan metabolism |
|  | C8H9NO3(+Na⁺) | [Pyridoxal +Na]⁺ | Vitamin B6 metabolism |
|  |  | [Isopyridoxal +Na]⁺ | Vitamin B6 metabolism |
|  |  | [3-Methoxyanthranilate +Na]⁺ | Tryptophan metabolism |
| 205.9977 | C3H9N3O3S(+K⁺) | [Taurocyamine+K]⁺ | Taurine and hypotaurine metabolism |
| 212.0317 | C10H7NO3(+Na⁺) | [N-Acetylisatin+Na]⁺ | Tryptophan metabolism |
|  |  | [4-Hydroxy-2-quinolinecarboxylic acid+Na]⁺ | Tryptophan metabolism |
|  | C7H11NO4(+K⁺) | [N-Acetyl-L-glutamate 5-semialdehyde+K]⁺ | Arginine and proline metabolism |
| 213.0352 | C7H10O6(+Na⁺) | [3-Dehydroquinate+Na]⁺ | Phenylalanine, tyrosine and tryptophan biosynthesis |
|  |  | [2,4-Dihydroxyhept-2-enedioate+Na]⁺ | Tyrosine metabolism |
| 228.0055 | C10H7NO3(+K⁺) | [N-Acetylisatin+K]⁺ | Tryptophan metabolism |
| 230.0422 | C5H12NO7P(+H⁺) | [5-Phosphoribosylamine+H]⁺ | Alanine, aspartate and glutamate metabolism |
|  | C10H9NO4(+Na⁺) | [2-Formaminobenzoylacetate+Na]⁺ | Tryptophan metabolism |
|  |  | [4-(2-Aminophenyl)-2,4-dioxobutanoate+Na]⁺ | Tryptophan metabolism |
|  | C7H13NO5(+K⁺) | [2-Amino-3,7-dideoxy-D-threo-hept-6-ulosonic acid+K]⁺ | Phenylalanine, tyrosine and tryptophan biosynthesis |

**References:**

(1) Horai, H.; Arita, M.; Kanaya, S.; Nihei, Y.; Ikeda, T.; Suwa, K.; Ojima, Y.; Tanaka, K.; Tanaka, S.; Aoshima, K.; Oda, Y.; Kakazu, Y.; Kusano, M.; Tohge, T.; Matsuda, F.; Sawada, Y.; Hirai, M. Y.; Nakanishi, H.; Ikeda, K.; Akimoto, N.; Maoka, T.; Takahashi, H.; Ara, T.; Sakurai, N.; Suzuki, H.; Shibata, D.; Neumann, S.; Iida, T.; Tanaka, K.; Funatsu, K.; Matsuura, F.; Soga, T.; Taguchi, R.; Saito, K.; Nishioka, T. MassBank: A Public Repository for Sharing Mass Spectral Data for Life Sciences. *J. Mass Spectrom.* **2010**, *45* (7), 703–714. https://doi.org/10.1002/jms.1777.

(2) Smith, C. A.; Maille, G. O.; Want, E. J.; Qin, C.; Trauger, S. A.; Brandon, T. R.; Custodio, D. E.; Abagyan, R.; Siuzdak, G. METLIN: A Metabolite Mass Spectral Database. *Ther. Drug Monit.* **2005**, *27* (6), 747–751. https://doi.org/10.1097/01.ftd.0000179845.53213.39.

(3) Leader, D. P.; Burgess, K.; Creek, D.; Barrett, M. P. Pathos: A Web Facility That Uses Metabolic Maps to Display Experimental Changes in Metabolites Identified by Mass Spectrometry. *Rapid Commun. Mass Spectrom.* **2011**, *25* (22), 3422–3426. https://doi.org/10.1002/rcm.5245.
